# GnRH Deficient Patients With Congenital Hypogonadotropic Hypogonadism: Novel Genetic Findings in *ANOS1, RNF216, WDR11, FGFR1, CHD7*, and *POLR3A* Genes in a Case Series and Review of the Literature

**DOI:** 10.3389/fendo.2020.00626

**Published:** 2020-08-28

**Authors:** Vassos Neocleous, Pavlos Fanis, Meropi Toumba, George A. Tanteles, Melpo Schiza, Feride Cinarli, Nicolas C. Nicolaides, Anastasis Oulas, George M. Spyrou, Christos S. Mantzoros, Dimitrios Vlachakis, Nicos Skordis, Leonidas A. Phylactou

**Affiliations:** ^1^Department of Molecular Genetics, Function and Therapy, The Cyprus Institute of Neurology and Genetics, Nicosia, Cyprus; ^2^Cyprus School of Molecular Medicine, The Cyprus Institute of Neurology and Genetics, Nicosia, Cyprus; ^3^Pediatric Endocrine Clinic, IASIS Hospital, Paphos, Cyprus; ^4^Clinical Genetics Department, The Cyprus Institute of Neurology and Genetics, Nicosia, Cyprus; ^5^Division of Endocrinology, Diabetes and Metabolism, First Department of Pediatrics, National and Kapodistrian University of Athens Medical School, “Aghia Sophia” Childrens Hospital, Athens, Greece; ^6^Division of Endocrinology and Metabolism, Biomedical Research Foundation of the Academy of Athens, Athens, Greece; ^7^Bioinformatics ERA Chair, The Cyprus Institute of Neurology and Genetics, Nicosia, Cyprus; ^8^Division of Endocrinology, Diabetes and Metabolism, Beth Israel Deaconess Medical Center, Harvard Medical School, Boston, MA, United States; ^9^Section of Endocrinology, Diabetes and Metabolism, Boston VA Healthcare System, Boston, MA, United States; ^10^Laboratory of Genetics, Department of Biotechnology, School of Food, Biotechnology and Development, Agricultural University of Athens, Athens, Greece; ^11^Lab of Molecular Endocrinology, Center of Clinical, Experimental Surgery and Translational Research, Biomedical Research Foundation of the Academy of Athens, Athens, Greece; ^12^Department of Informatics, Faculty of Natural and Mathematical Sciences, King's College London, London, United Kingdom; ^13^Division of Pediatric Endocrinology, Paedi Center for Specialized Pediatrics, Nicosia, Cyprus; ^14^St George's, University of London Medical School at the University of Nicosia, Nicosia, Cyprus

**Keywords:** GnRH, hypogonadotropic hypogonadism, genes, digenic inheritance, next generation sequencing

## Abstract

**Background:** Congenital hypogonadotropic hypogonadism (CHH) is a rare genetic disease caused by Gonadotropin-Releasing Hormone (GnRH) deficiency. So far a limited number of variants in several genes have been associated with the pathogenesis of the disease. In this original research and review manuscript the retrospective analysis of known variants in *ANOS1* (*KAL1), RNF216, WDR11, FGFR1, CHD7*, and *POLR3A* genes is described, along with novel variants identified in patients with CHH by the present study.

**Methods:** Seven GnRH deficient unrelated Cypriot patients underwent whole exome sequencing (WES) by Next Generation Sequencing (NGS). The identified novel variants were initially examined by *in silico* computational algorithms and structural analysis of their predicted pathogenicity at the protein level was confirmed.

**Results:** In four non-related GnRH males, a novel X-linked pathogenic variant in *ANOS1* gene, two novel autosomal dominant (AD) probably pathogenic variants in *WDR11* and *FGFR1* genes and one rare AD probably pathogenic variant in *CHD7* gene were identified. A rare autosomal recessive (AR) variant in the *SRA1* gene was identified in homozygosity in a female patient, whilst two other male patients were also, respectively, found to carry novel or previously reported rare pathogenic variants in more than one genes; *FGFR1*/*POLR3A* and *SRA1/RNF216*.

**Conclusion:** This report embraces the description of novel and previously reported rare pathogenic variants in a series of genes known to be implicated in the biological development of CHH. Notably, patients with CHH can harbor pathogenic rare variants in more than one gene which raises the hypothesis of locus-locus interactions providing evidence for digenic inheritance. The identification of such aberrations by NGS can be very informative for the management and future planning of these patients.

## Introduction

Congenital hypogonadotropic hypogonadism (CHH) is a rare disorder that is mainly caused by gonadotropin releasing hormone (GnRH) deficiency and characterized by delayed sexual development and infertility in both males and females (1–6). The pulsatile secretion of the decapeptide GnRH from the hypothalamus into the hypophyseal-portal vessels exerts control in the synthesis and release of luteinizing hormone (LH) and follicle stimulating hormone (FSH) in the anterior pituitary gland (7, 8). In CHH, the GnRH secretion and/or action is impaired and as a consequence patients with the disorder exhibit low levels of gonadotropins, low sex steroids, absent, incomplete or delayed puberty and subsequently hypogonadotropic hypogonadism (HH) (1, 9, 10). The prevalence of CHH is estimated to be 1:8,000 male and 1:40,000 female live births with slightly fewer than 50% of cases suffering from hyposmia or anosmia (10–12). CHH is divided into two subtypes, which include of congenital normosmic isolated hypogonadotropic hypogonadism (HH) and anosmic HH or Kallmann syndrome (KS) (13, 14). Current research regarding the pathophysiology of CHH provides evidence that genetic abnormalities play a key role in the development of the disease and is estimated that a genetic cause is apparent in almost 50% of CHH cases (1, 6). Up-to-date there have been reported more than 60 putative loci for CHH, 17 of which have been linked with KS (1, 6, 13–16).

Over the last few years with the use of the high throughput next generation sequencing (NGS) the number of genes shown to be responsible for causing CHH/KS has radically increased (15, 17). Therefore, the purpose of the present study was to determine the genetic involvement in a series of clinically diagnosed with CHH/KS Cypriot patients.

## Materials and Methods

### Patients

A total of seven (six males and one female) unrelated Cypriot patients with CHH/KS were included in the present study and underwent whole exome sequencing by NGS. Clinical criteria included the absence or incomplete development of secondary sexual characteristics after the age of 16 years in females and 18 years in males. The biochemical criteria included low levels of basal and GnRH stimulated gonadotrophins (LH, FSH) as well as low levels of sex steroids (testosterone in males and estradiol in females). MRI scans were performed for all patients, with the exception of patients 2 and 6. Five male patients and one female exhibited isolated hypogonadotropic hypogonadism. Only one patient, a 72-year-old male, had KS with anosmia. Written, informed consent was obtained from all seven adult individuals that participate in the study for the publication of any potentially identifiable images or data included in this article. The study was approved by the Cyprus National Bioethics Committee and all methods were performed in accordance with the relevant guidelines and regulations.

### Genetic Analysis

Genomic DNA was isolated from peripheral blood using the Gentra Puregene Kit (Qiagen, Valencia, CA, USA) according to the manufacturer's instructions. The DNA concentration and purity was measured using the Nanodrop ND-1000 spectrophotometer (NanoDrop Technologies, Wilmington, DE, USA). Prior to library preparation for whole exome sequencing (WES) genomic DNA was quantified using the Qubit dsDNA BR Assay Kit (Invitrogen, Life Technologies, Eugene, OR, USA) on a Qubit® 2.0 Fluorometer (Invitrogen, Life Technologies, Eugene, OR, USA). WES was performed by using the TruSeq Exome Kit (Illumina Inc., San Diego, CA, USA) with paired-end 150 bp reads. NGS was performed using the NextSeq 500/550 High Output Kit v2.5 (150 Cycles) on an NextSeq500 system (Illumina Inc., San Diego, CA, USA). The FastQC quality control tool (http://www.bioinformatics.babraham.ac.uk/projects/fastqc/) was used to evaluate the quality of the WES procedure. The mean target coverage for the exome was 70.88X. Specifically, 10X coverage was achieved for 99.22% of the nucleotides, 20X coverage for 87.68% of the nucleotides and 30X coverage for 79.35% of the nucleotides, indicating that the WES reaction was of sufficiently high quality for subsequent analysis.

### Variant Analysis

The fastqc data obtained by WES were processed using an in-house bioinformatics pipeline. Briefly, all variants were inputted into the VarApp Browser and filtered. VarApp is a graphical user interface, which supports GEMINI (18). Variants in selected genes with biological involvement in the GnRH neuronal system and CHH (Supplementary Table 1) were further analyzed using the Qualimap v2.2.1 tool (19) to calculate the target coverage. Mean target coverage was >20X for 93.2% of the selected genes and >30X for 89% of the selected genes (Supplementary Table 1). Variants in these genes were additionally filtered using the VarApp Browser for minor allele frequencies of <1% in public databases such as 1000 genomes, ExAC browser and Exome Sequencing Project (ESP). Moreover, variants were filtered and selected according to their impact such as frameshift, splice acceptor, splice donor, start lost, stop gained, stop lost, inframe deletion, inframe insertion, missense, protein altering and splice region. In addition, variants were filtered by the VarApp Browser for their pathogenicity by two *in silico* tools, SIFT and Polyphen2. Population-specific data from an in-house WES library composed of 51 randomly selected samples of Cypriot origin were used to evaluate the potential disease-causing variants. All variants identified were confirmed by Sanger sequencing. When genetic material of relatives was obtainable, familial segregation was performed (Table 2, Supplementary Figure 5). For the cases where two potentially pathogenic variants were identified in an individual, we employed the ORVAL platform for predicting pathogenicity due to digenicity (20). Finally, the variants were categorized for their pathogenicity using the standards and guidelines of the American College of Medical Genetics and Genomics and the Association for Molecular Pathology (21).

### *In silico* Analysis of the Single Nucleotide Variants

*In silico* prediction on protein function of the pathogenicity effect of the different amino acid substitutions identified by NGS and confirmed with Sanger analysis was performed by the PredictSNP tool using the default settings (22). The PredictSNP tool evaluates the pathogenicity of a variant by using seven different protein functionality prediction tools: MAPP, PhD-SNP, PolyPhen-1, PolyPhen-2, SIFT, SNAP, and PANTHER.

### Molecular Modeling and Homology Modeling of the Mutated Genes

By using MOE (Chemical Computing Group, MOE, v2014.0901, www.chemcomp.com) homology modeling was attempted in all seven genes that carried the missense variants identified in the present study. The selection of template crystal structures for homology modeling was based on the primary sequence identity and the crystal resolution. The MOE homology model method is separated into four main steps. First, a primary fragment geometry specification. Second the insertion and deletions task. The third step is the loop selection and the side-chain packing and the last step is the final model selection and refinement. The template selection was as follows: for PROP1 the 3A01 PDB file was used, for SRA1 the 4NBO PDB file was used, for RNF216 the 5l1V PDB file was used, for FGFR1 the 1CVS PDB file was used and for POLR3A the 5FJ8 PDB file was used. All models were handled, verified and visualized using the Drugster suite (23).

### Model Optimization

Energy minimization for all four models was done in MOE initially using the Amber99 (24) force-field implemented into the same package, up to a root mean square deviation (RMSd) gradient of 0.0001 to remove the geometrical strain. The models were subsequently solvated with simple point charge (SPC) water using the truncated octahedron box extending to 7 Å from the model, and molecular dynamics was performed at 300 K, 1 atm with 2 fs step size for a total of 10 ns, using the NVT ensemble in a canonical environment (NVT stands for Number of atoms, Volume and Temperature that remain constant throughout the calculation). The results of the molecular dynamics simulation were collected into a database by MOE for further analysis.

## Results

### Genetic Findings

**Figure 1:**
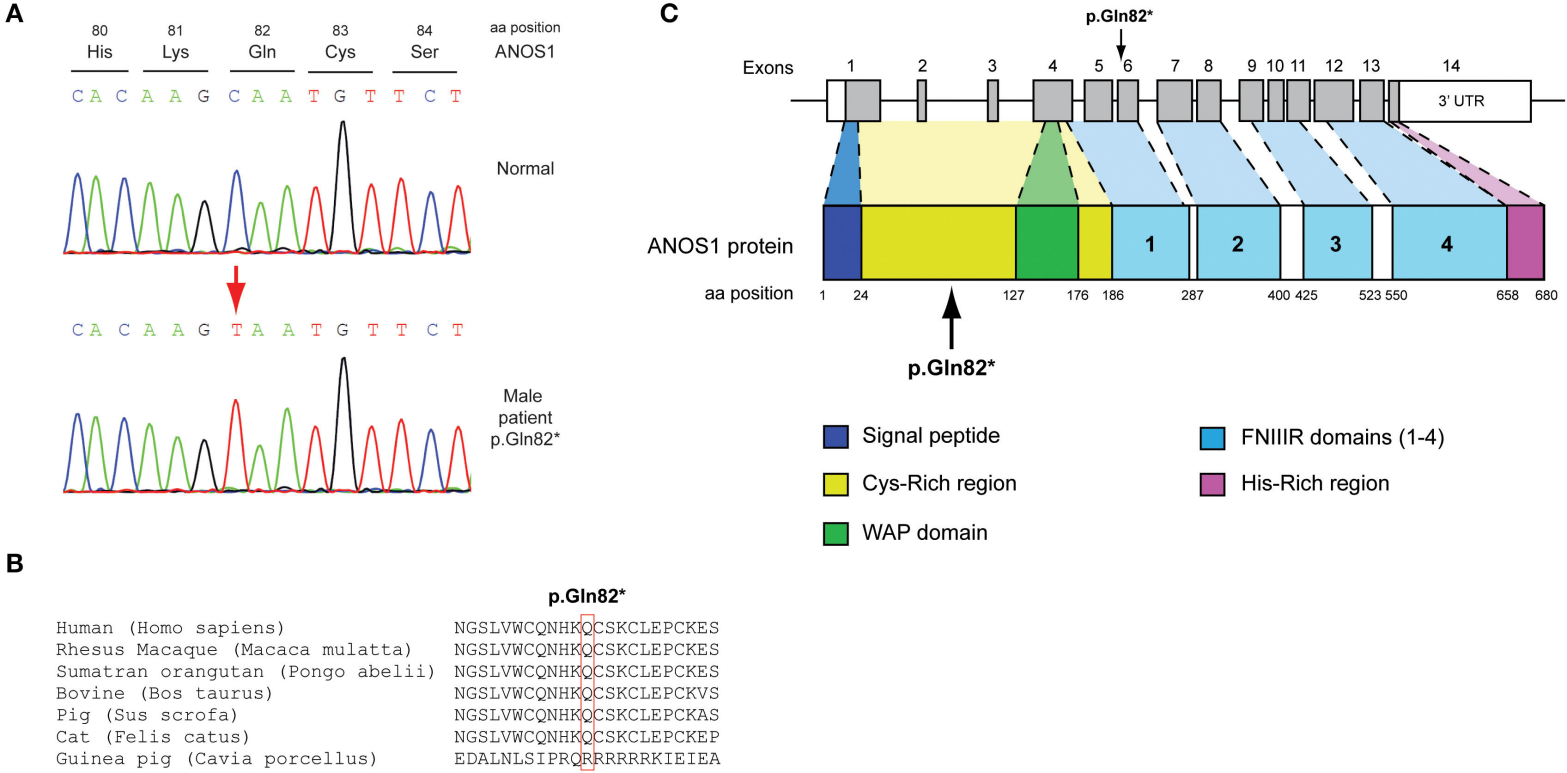
Identification of a novel p.Gln82* *ANOS1* mutation. **(A)** Sequence electropherogram of the novel p.Gln82* *ANOS1* mutation. **(B)** Multiple sequence alignment of the amino acid at position 82 of the ANOS1 protein from various species. The conserved glutamine amino acid at position 82 is indicated by red color. **(C)** Schematic representation of the *ANOS1* gene and protein of a male patient identified with the novel p.Gln82* non-sense pathogenic variant. WAP, whey acidic protein; FNIII, fibronectin type III.

**Figure 2:**
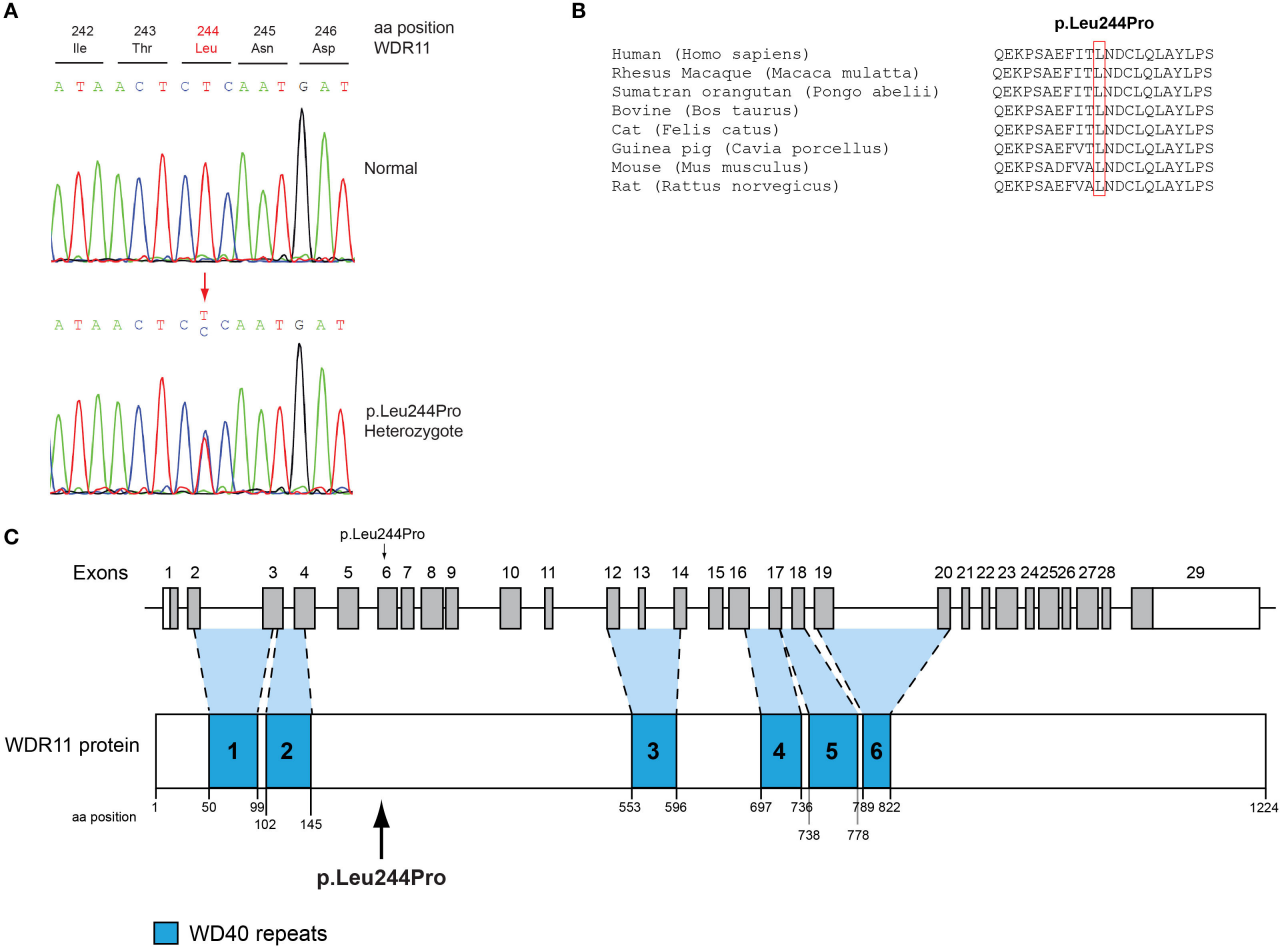
Identification of a novel p.Leu244Pro *WDR11* variant. **(A)** Sequence electropherogram of the novel *WDR11* p.Leu244Pro variant. **(B)** Multiple sequence alignment of the amino acid at position 244 of the WDR11 protein from various species. The conserved leucine amino acid at position 244 is indicated by red color. **(C)** Schematic representation of the *WDR11* gene and protein of a male patient identified with the novel p.Leu244Pro variant.

**Figure 3:**
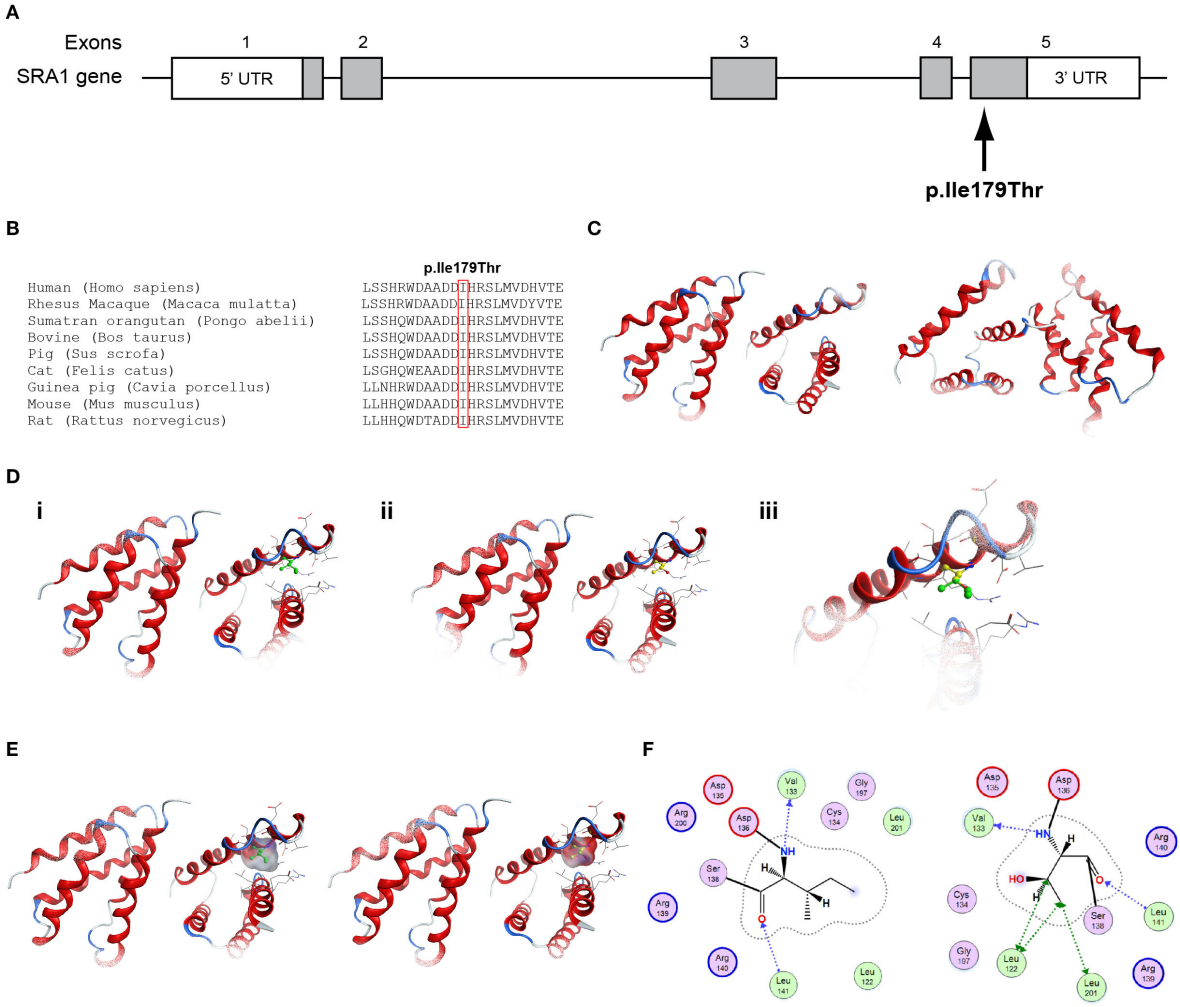
Identification of the p.Ile179Thr *SRA1* variant. **(A)** Schematic representation of the *SRA1* gene. The known *SRA1* p.Ile179Thr variant identified in a male patient in the heterozygote state and in a female patient in the homozygous state is indicated. **(B)** Multiple sequence alignment of the amino acid at position 179 of the SRA1 protein from various species. The conserved isoleucine amino acid at position 179 is indicated by red color. **(C)** The homology model for SRA1. **(D)** Design of the wild type (i) and mutant model (ii) of SRA1. (iii) Wild type and mutant models superposed. **(E)** Electrostatic surface calculated and drawn for the wild type and mutant models. **(F)** 2D interaction diagram for wild type and mutant models.

All seven patients were sequenced by WES. The clinical, biochemical and genetic characteristics are summarized in Table 1. A total of nine variants were identified in genes that are known to be linked with the development of CHH/KS (Table 2). Seven of these variants were novel and two were previously reported. The novel X-linked p.Gln82^*^ in the *ANOS1 (KAL1)* gene was found in patient 1, a 28-year-old CHH male with pubertal absence, cryptorchidism and micropenis (Table 1, Figure 1). The novel *WDR11* p.Leu244Pro variant is probably pathogenic and is inherited in an autosomal dominant fashion (AD). This variant was identified in patient 2, a 72-year-old male with KS and associated clinical characteristics of anosmia, cryptorchidism and micropenis. Patient 2 first sought medical advice at the age of 40 years and since then remains a patient of our clinic. Molecular diagnosis was only possible 32 years later (Table 1, Figure 2). The previously reported AR, probably pathogenic p.Ile179Thr variant in the *SRA1* gene was identified in heterozygosity in patient 3, a 19-year-old male with partial hypogonadism and upper limb defects (Table 1, Figure 3). In addition, the novel p.Asp792Asn in the *RNF216* gene was also identified in heterozygosity in the same patient (Figures 3, 8, Supplementary Figure 1). Evaluation by the ORVAL platform for digenicity predicted this novel variant to have a neutral effect (Supplementary Figure 6). However, familial segregation data and *in silico* structural models indicated a digenic mode of inheritance (Figures 3, 8, Supplementary Figure 5). Variants in the *SRA1* and *RNF216* genes have been associated with effects on the CHH phenotype. Thus, the presence in patient 3 of pathogenic, heterozygous variants in *SRA1* and *RNF216* genes could potentially be another example of digenic inheritance for the development of CHH (28).

**Table 1:** Clinical and biochemical characteristics of the patients with CHH.

	**Patient 1**	**Patient 2**	**Patient 3**	**Patient 4**	**Patient 5**	**Patient 6**	**Patient 7**
Current age	28-years	72-years	19-years	18-years	31-years	20-years	30-years
Sex	Male	Male	Male	Male	Male	Male	Female
Main Phenotype	CHH	KS	CHH	CHH	CHH	CHH	CHH
Associated clinical characteristics	Cryptorchidism; micropenis	Cryptorchidism; micropenis; gynaecomastia; Anosmia	-	Cryptorchidism; micropenis	Cryptorchidism; micropenis	Cryptorchidism; micropenis; gynaecomastia	N/A
Partial or absent puberty	Absent	Absent	Partial (Tanner stage 3)	Absent	Absent	Absent	Absent
Overlapping syndromes	NO	NO	Upper limb defects	NO	Hypodontia and hypogonadotropic hypogonadism	NO	NO
GnRH Reversal	NO	NO	YES	NO	NO	NO	NO
CHH Sex Reversal	NO	NO	NO	NO	NO	NO	NO
Gene(s)	*ANOS1*	*WDR11*	*SRA1^#^/RNF216^^¥^^*	*CHD7*	*FGFR1^$^/POLR3A^€^*	*FGFR1*	*SRA1*
Genotype	p.Gln82*	p.Leu244Pro/WT	p.Ile179Thr^#^/WT; p.Asp792Asn^^¥^^/WT	p.Arg2400Trp/WT	p.Pro186Ala*^$^*/WT; p.Arg561Gly^€^/WT	p.Arg822Cys/WT	p.Ile179Thr/p.Ile179Thr
Mode of Inheritance	X-linked	AD	AR^#^/AR^^¥^^ (Digenic)	AD	AD^$^ (due to the pathogenic variant in *FGFR1)*	AD	AR
LH <2 IU/L	0.10	n.d.	0.13	0.09	0.11	0.1	n.d.
FSH <2 IU/L	0.10	n.d.	n.d.	0.5	0.13	0.5	0.1
Testosterone nmol/L <1	0.08	n.d.	0.4	0.2	1.1	1.3	-
Testicular Volume (ml)	3.0	3.0	5.0	3.0	2.0	4.0	N/A
Ovarian Volume (cm^3^)	N/A	N/A	N/A	N/A	N/A	N/A	1.0
Primary amenorrhea	N/A	N/A	N/A	N/A	N/A	N/A	Yes
MRI	Normal	N/A	Normal	Normal	Normal	N/A	Normal

*AD, Autosomal Dominant; AR, Autosomal Recessive; n.d., not detectable; N/A, Not Applicable*;

# *The p.Ile179Thr variation of the SRA1 gene and its associated inheritance*;

¥ *The p.Asp792Asn variation of the RNF216 gene and its associated inheritance*;

$ *The p.Pro186Ala variation of the FGFR1 gene and its associated inheritance*;

€ *The p.Arg561Gly variation of the POLR3A gene*.

**Table 2:** Variants identified in the seven non-related patients.

**Patient ID**	**Sex**	**Phenotype**	**Gene**	**Refseq**	**Variant identified (cDNA)**	**Variant identified (protein)**	**Genotype**	**Inheritance**	**MAF (%)—gnomAD v2.1.1**	**Population Specific frequency (51 WES random samples)**	**Familial Segregation**	**Variant classification**	**Previously described**	**Additional genetic variants**
1	M	CHH	*ANOS1*	NM_000216.4:c.244C>T	c.244G>A	p.Gln82*	p.Gln82*	X-linked	Absent	Absent	YES	Pathogenic	-	-
2	M	KS	*WDR11*	NM_018117.12:c.731T>C	c.731T>C	p.Leu244Pro	p.Leu244Pro/WT	AD	Absent	Absent	NA	Probably Pathogenic	-	-
3	M	CHH	*SRA1*	NM_001035235.3:c.536T>C	c.536T>C	p.Ile179Thr	p.Ile179Thr/WT	AR	0.00081	Absent	YES	Probably Pathogenic	(25, 26)	MC4R: p.Val103Ile/WT
			*RNF216*	NM_207111.3:c.2374G>A	c.2374G>A	p.Asp792Asn	p.Asp792Asn/WT	AR	Absent	Absent		Probably Pathogenic	-	
4	M	CHH	*CHD7*	NM_017780.4:c.7198C>T	c.7198C>T	p.Arg2400Trp	p.Arg2400Trp/WT	AD	0.0000154	Absent	NA	Probably Pathogenic	-	PROP1: p.Arg112Gln/WT (MAF: 0.000255%) MC4R: p.Val103Ile/WT
5	M	CHH	*FGFR1*	NM_023110.3:c.556C>G	c.556C>G	p.Pro186Ala	p.Pro186Ala/WT	AD	Absent	Absent	NA	Probably Pathogenic	-	–
			*POLR3A*	NM_007055.4:c.1681C>G	c.1681C>G	p.Arg561Gly	p.Arg561Gly/WT	AR	Absent	Absent		Probably Pathogenic	-	
6	M	CHH	*FGFR1*	NM_023110.3:c.2464C>T	c.2464C>T	p.Arg822Cys	p.Arg822Cys/WT	AD	0.00026	Absent	NA	Probably Pathogenic	(27)	-
7	F	CHH	*SRA1*	NM_001035235.3:c.536T>C	c.536T>C	p.Ile179Thr	p.Ile179Thr/p.Ile179Thr	AR	0.00081	Absent	NA	Probably Pathogenic	(25, 26)	-

*KS, Kallman syndrome; CHH, Congenital Hypogonadotropic Hypogonadism; WT, Wild Type; AD, Autosomal Dominant; AR, Autosomal Recessive; MAF, Minor Allele Frequency; gnomAD, Genome Aggregation Database (https://gnomad.broadinstitute.org/); WES, Whole Exome Sequencing; NA, Not Available*.

**Figure 4:**
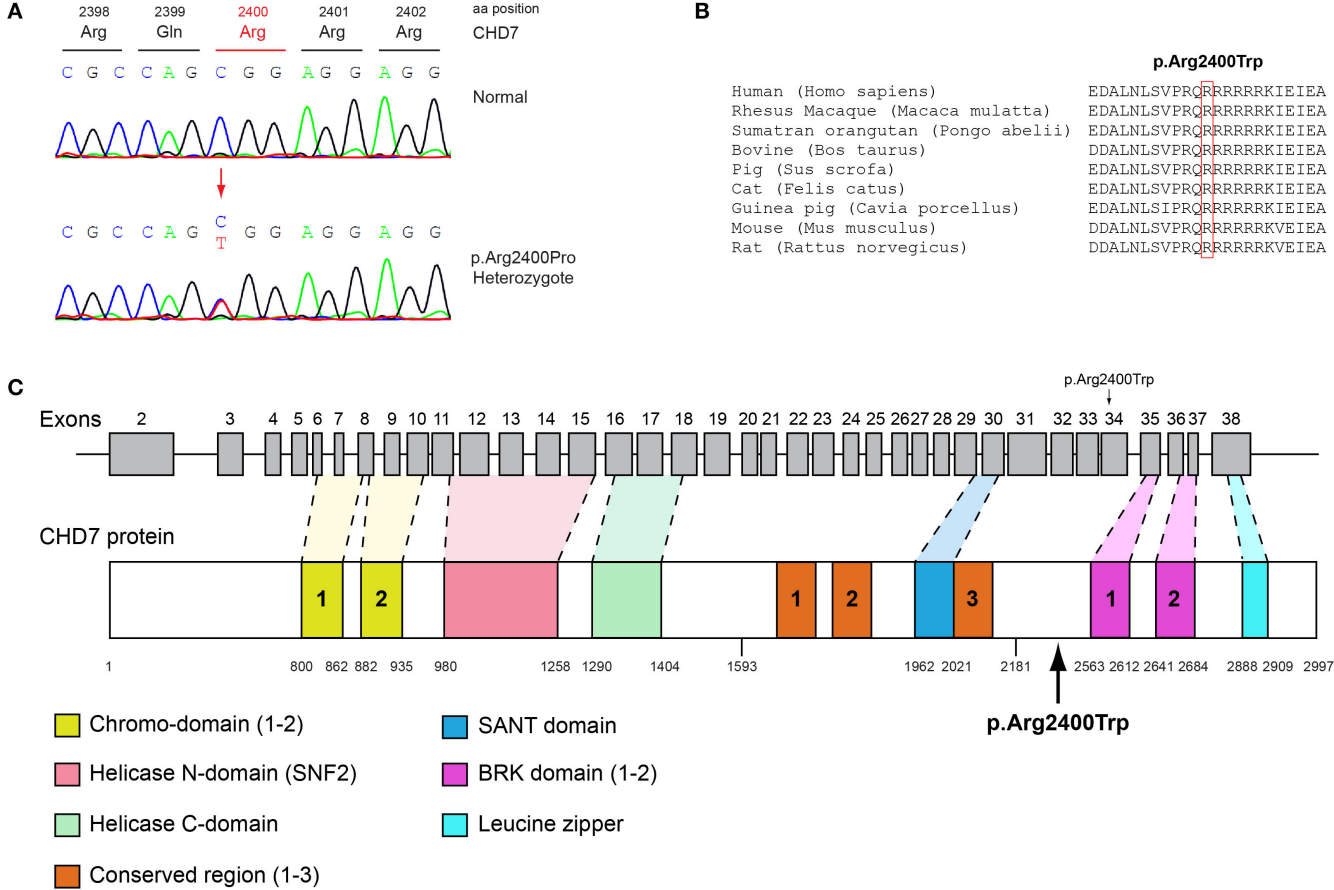
Identification of the p.Arg2400Trp *CHD7* variant. **(A)** Sequence electropherogram of a male patient identified with the novel *CHD7* p.Arg2400Trp variant. **(B)** Multiple sequence alignment of the amino acid at position 2,400 of the CHD7 protein from various species. The conserved arginine amino acid at position 2,400 is indicated by red color. **(C)** Schematic representation of the *CHD7* gene and protein of a male patient identified with the novel p.Arg2400Trp variant.

**Figure 5:**
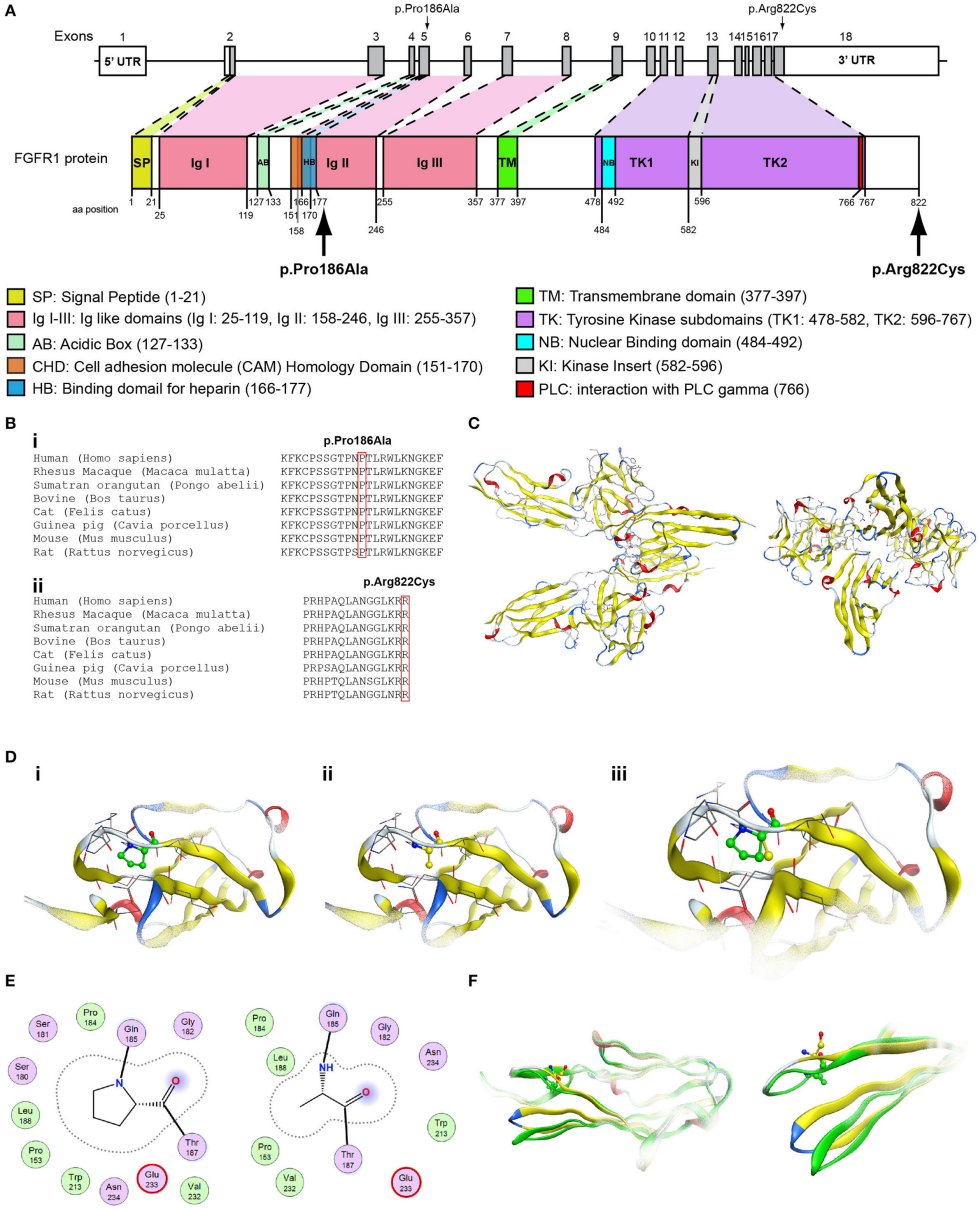
Identification of the p.Pro186Ala and p.Arg822Cys *FGFR1* variants. **(A)** Schematic representation of the *FGFR1* gene and protein. The p.Pro186Ala and p.Arg822Cys *FGFR1* variants are indicated with arrows. **(B)** Multiple sequence alignment of the amino acids at position 186 (i) and 822 (ii) of the FGFR1 protein from various species. The conserved amino acids at positions 186 and 822 are indicated by red color. **(C)** The homology model of the FGFR1. **(D)** Design of the wildtype (i) and p.Pro186Ala mutant model (ii); the wildtype and p.Pro186Ala mutant models superposed (iii). **(E)** 2D interaction diagram for wildtype and p.Pro186Ala mutant models. **(F)** Conformational change induced upon variant. Wildtype is showing in green ribbon, while p.Pro186Ala mutant is showing in yellow ribbon.

**Figure 6:**
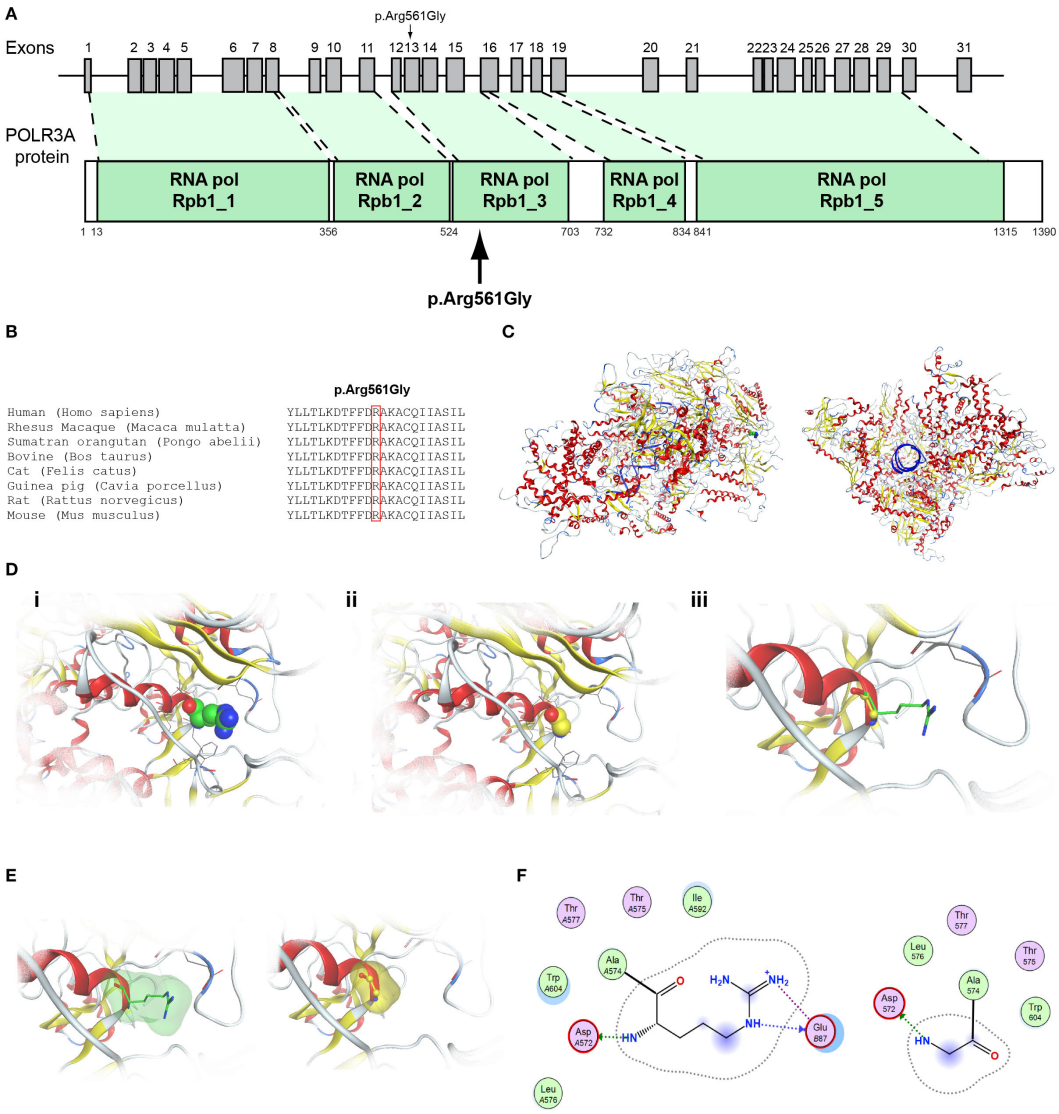
Identification of the p.Arg561Gly *POLR3A* variant. **(A)** Schematic representation of the *POLR3A* gene and protein. The p.Arg561Gly *POLR3A* variant is indicated with arrows. **(B)** Multiple sequence alignment of the amino acid at position 561 of the POLR3A protein from various species. The conserved arginine amino acid at position 561 is indicated by red color. **(C)** The homology model for POLR3A. **(D)** Design of the wild type (i) and mutant model (ii) of POLR3A. (iii) Wild type and mutant models superposed. **(E)** Electrostatic surface calculated and drawn for the wild type and mutant models. **(F)** 2D interaction diagram for wild type and mutant models.

Patient 4, an 18-year-old male with CHH and the associated clinical characteristics of cryptorchidism and micropenis, was found to carry the novel AD p.Arg2400Trp variant in the *CHD7* gene (Table 1, Figure 4). Patient 5, a 31-year-old male with CHH and associated clinical characteristics of cryptorchidism and micropenis, was found to carry the novel AD p.Pro186Ala in the *FGFR1* gene (Table 1, Figure 5). The previously reported AD p.Arg822Cys also in the *FGFR1* gene was found in patient 6, a 20-year-old male with CHH and associated clinical characteristics of cryptorchidism, micropenis and gynaecomastia (Table 1, Figure 5, Supplementary Figure 2). The known p.Ile179Thr variant in the *SRA1* gene was also identified in the homozygous state in patient five, a 30-year-old female with CHH and primary amenorrhea (Figure 3, Supplementary Figure 1). Furthermore, patient five also carried the novel p.Arg561Gly variant in the *POLR3A* gene (Figure 6, Supplementary Figure 3). The ORVAL platform was used to evaluate the pathogenicity of the variants regarding digenic inheritance, with respect to which they were shown to be positive (Supplementary Figure 6). Such variants when inherited in the AR form are usually associated with Pol III-related hypomyelinating leukodystrophies and not with CHH. Patients with Pol III-related leukodystrophies may have various clinical characteristics including ataxia, delayed dentition, hypomyelination, hypodontia, and hypogonadotropic hypogonadism. Patient 5, in addition to CHH, also developed a mild hypomyelinating leukodystrophy phenotype, which is likely associated with the heterozygous condition found in the *POLR3A* gene.

**Figure 7:**
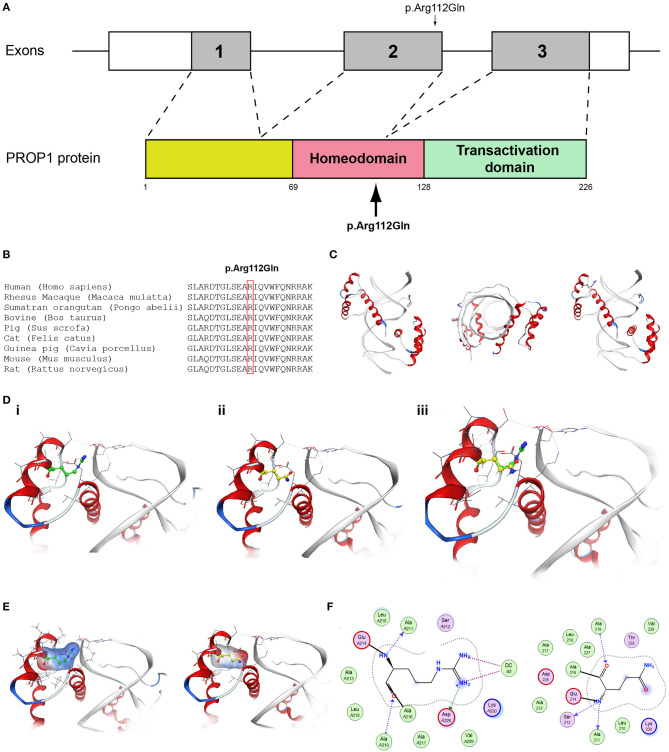
Identification of the p.Arg112Gln *PROP1* variant. **(A)** Schematic representation of the PROP1 gene and protein. The p.Arg112Gln *PROP1* variant is indicated with arrows. **(B)** Multiple sequence alignment of the amino acid at position 112 of the PROP1 protein from various species. The conserved arginine amino acid at position 112 is indicated by red color. **(C)** The homology model for PROP1. **(D)** Design of the wild type (i) and mutant model (ii) of PROP1. (iii) Wild type and mutant models superposed. **(E)** Electrostatic surface calculated and drawn for the wild type and mutant models. **(F)** 2D interaction diagram for wild type and mutant models.

It should also be noted that 3 of our patients were found to have other variants as well. The known p.Val103Ile variation of the *MC4R* gene, which has been linked to obesity, was found in heterozygocity in two of the male patients (the 18 and the 19-year-old males, Patients two and four). Both the patients were obese, with BMI above +2SDS and both developed insulin resistance. In addition the novel variant p.Arg112gGln in the *PROP1* gene was found in the heterozygous state also in patient 4, an 18-year-old male (Figure 7, Supplementary Figure 4). Such variants have been reported to be associated with combined pituitary hormone deficiency. Our patient also had central hypothyroidism and he is currently on treatment with thyroxin.

The novel non-sense pathogenic variant p.Gln82^*^ in the *ANOS1* gene, which was identified in a 28-year-old male, encodes a premature termination codon. It is expected to yield a truncated ANOS1 protein, missing the whey acidic protein (WAP)-like protease inhibitor domain and the four fibronectin type III (FN[III]) domains (Figure 1). The missense variants, identified by WES and confirmed by Sanger sequencing, were predicted to be deleterious by at least two prediction tools using the PredictSNP consensus classifier (22) (Table 3). The PredictSNP consensus classifier evaluates the pathogenicity of a variant by using seven different prediction tools: MAPP, PhD-SNP, PolyPhen-1, PolyPhen-2, SIFT, SNAP, and PANTHER. Furthermore, the identified variants were absent from the population-specific data in an in-house database composed of 51 random samples of Cypriot origin. Familial segregation was available for two patients, patient 1 and patient 3 (Supplementary Figure 5, Table 2). For patient one the identified pathogenic variant followed the X-liked mode of inheritance and for patient 3, variants followed the digenic mode of inheritance (Supplementary Figure 5).

**Table 3:** Prediction analysis of the variants identified.

**Gene**	**Variant**	**PredictSNP (%)**	**MAPP (%)**	**PhD-SNP (%)**	**PolyPhen-1 (%)**	**PolyPhen-2 (%)**	**SIFT(%)**	**SNAP (%)**	**PANTHER (%)**
*WDR11*	p.Leu244Pro	61	77	68	67	56	79	50	69
*SRA1*	p.Ile179Thr	61	81	59	67	63	79	50	-
*CHD7*	p.Arg2400Trp	64	-	78	74	81	45	62	-
*FGFR1*	p.Pro186Ala	61	72	86	67	59	79	72	-
	p.Arg822Cys	72	-	66	74	81	79	72	-
*POLR3A*	p.Arg561Gly	87	48	82	74	50	79	62	72
*PROP1*	p.Arg112Gln	87	41	88	74	81	79	72	-

*Percentage of confidence is shown by various prediction methods under the PredictSNP tool. Neutral and deleterious prediction effects are shown in green and red, respectively*.

### Conserved Protein Sequences Among Species

**Figure 8:**
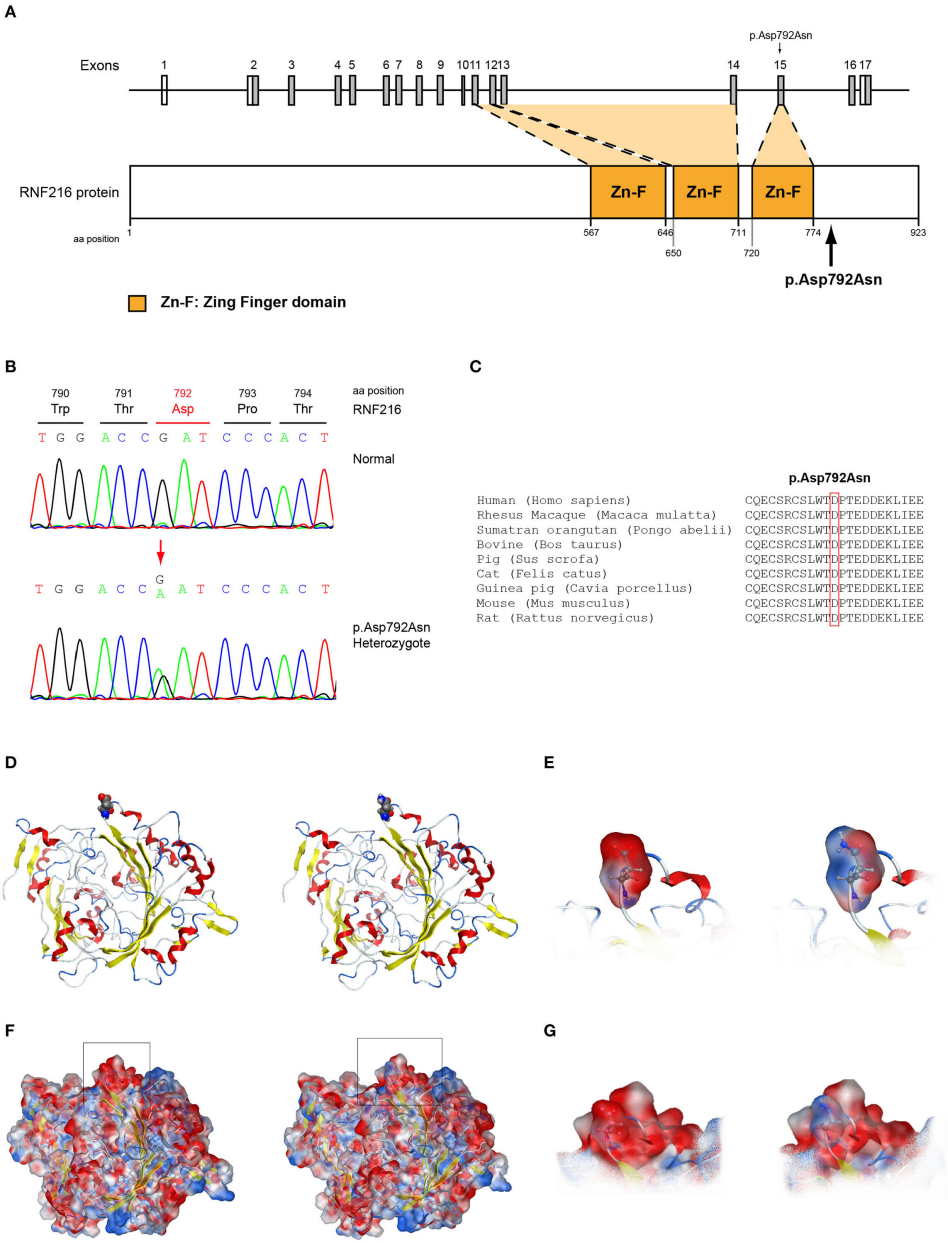
Identification of the p.Asp792Asn *RNF216* variant**. (A)** Schematic representation of the *RNF216* gene and protein of a male patient identified with the novel p.Asp792Asn variant. **(B)** Sequence electropherogram of a male patient identified with the novel *RNF216* p.Asp792Asn variant. **(C)** Multiple sequence alignment of the amino acid at position 792 of the RNF216 protein from various species. The conserved aspartic acid amino acid at position 792 is indicated by red color. **(D)** The p.Asp792Asn *RNF216* variant is indicated with spacefill atoms. **(E)** The electrostatic potential surface calculated and drawn for the wild type and mutant residues at the 792 position of *RNF216*. **(F)** The electrostatic potential surface calculated and drawn for the whole protein. In squares the regions presented in **(G)**. **(G)** The electrostatic potential surface calculated and drawn for the adjacent to the 792 position residues.

Protein alignment analyses of all identified pathogenic and probably pathogenic variants including the p.Gln82^*^ of the *ANOS1* gene, the p.Leu244Pro of the *WDR11* gene, the p.Asp792Asn of the *RNF216* gene, the p.Arg2400Trp of the *CHD7* gene, the p.Pro186Ala and p.Arg822Cys of the *FGFR1* gene, the p.Ile179Thr of the *SRA1* gene, the p.Arg561Gly of the *POLR3A* gene and the p.Arg112gGln of the *PROP1* gene showed high amino acid conservation among different species (Figures 1B, 2B, 3B, 4B, 5B, 6B, 7B, 8C). Therefore, the newly and previously discovered variant sites of the above genes were probably located in a vital region of the coding genes that might affect the corresponding proteins mechanistically and/or structurally.

### Effect of the Mutated *PROP1, SRA1, FGFR1, POLR3A*, and *RNF216* at the Related Protein Structure

The selection of template crystal structures for homology modeling was based on the primary sequence identity and crystal resolution models were obtained for the *PROP1, SRA1, FGFR1, POLR3A*, and *RNF216*. Unfortunately, no crystal resolution models were obtained for the CHD7 and WDR11 proteins.

The models for the *SRA1, FGFR1, POLR3A, PROP1*, and *RNF216* were designed using means of homology modeling (Figures 3C, 5C, 6C, 7C, 8D). Each one of the recorded variants were induced *in silico* and each model was energetically optimized via Energy minimizations and Molecular Dynamics [Figures 3D (i-ii), 5D (i-ii), 6D (i-ii), 7D (i-ii)]. The wild type and variant models were subsequently superposed and the variant residues were inevitably superposed too [Figures 3D (iii), 5D (iii), 6D (iii), 7D (iii)]. The electrostatic surface was calculated for each wild type and variant set for the SRA1, POLR3A, and PROP1 models (Figures 3E, 6E, 7E) and finally the 2D interaction diagram was drawn for each pair to visualize all bonding and conformation changes induced upon by the variants (Figures 3F, 5E, 6F, 7F). Since the FGFR1 variant involved the replacement of a proline residue the conformational change in the 3D conformation of the beta sheet formation is showed in Figure 5F.

More specifically, the p.Arg112Gln variant in the PROP1 results in a significant physicochemical change in the 112 position. The bulkier and positively charged arginine residue is now replaced by a smaller polar and uncharged glutamine residue. Inevitably the interaction to the DNA molecule that was also included in the model is now lost, as well as a strong stabilizing H-bond to a nearby negatively charged aspartic acid residue. It is evident from the 2D interaction map that the mutant PROP1 has lost its potential to interact with DNA and that would unavoidably alter its molecular and cellular function (Figure 7). Moving on, the p.Ile179Thr variant of the SRA1 also changes significantly the physicochemical profile of the amino acid at 179 position. Isoleucine is a non-polar aliphatic residue, whereas threonine is a polar residue. This electrostatic change results in the establishment of two new bonds with two nearby leucine residues. This fixates the local three helical bundle conformation more strongly, thus making the 3D structural arrangement adjacent to the 179 position more compact and conformationally rigid compared to the wild type (Figure 3). Likewise, the p.Pro186Ala variant in the FGFR1 has a significant conformational impact. The proline residue is a very special amino acid that bears a cyclic side chain. This gives this residue a unique property of inducing a kink in the 3D conformational arrangement of the protein. Removing it, and replacing it with an alanine residue resulted in a 2.5 Angstrom shift outwards of the beta sheet it is located in and that consequently led to a 4.5 Angstrom shift of the neighboring antiparallel beta sheet formation too. Such significant changes in structural arrangements are bound to change the mechanics and function of the FGFR1 protein (Figure 5). Lastly, the p.Arg561Gly in the POLR3A is a fine example of a variant change from a very bulky and positively charged residue to a small amino acid. From the 2D interaction diagram for this residue we can deduce that only the arginine amino acid is long enough to reach the nearby positively charged aspartic acid residue and to establish with the latter strong H-bonds. The glycine residue is not just small but it also misses the essential amino-groups that are required to establish H-bonding to the glutamic acid (Figure 6). The p.Asp792Asn variant according to the homology model of RNF216 is exposed to the solvent. It is located on a hairpin like loop, linking a beta-sheet and an a-helix in an antiparallel fashion (Figure 8D). Therefore, there is high probability that this could be an interacting part of the RNF216 protein, judging from the rotamer of this residue, which is pointing outwards. In this direction, we modeled the electrostatic potential of the 792 residue position (Figure 8E), of the adjacent residues within a 7 Angstrom radius (Figure 8G) and of the whole RNF216 protein (Figure 8F). It was found that the variation of aspartic acid to asparagine changes significantly the electrostatic potential and nature of the 792 position due to the extra NH2 moiety on the asparagine amino acid. That, coupled with the fact that all amino acids around the 792 position are negatively charged (red color–Figure 8G, left) is very significant as with the introduction of the asparagine residue a positively charged group is now introduced. Taking the abovementioned facts into consideration, we propose that the p.Asp792Asn variant is very significant as the positively charged moiety that is introduced could disrupt the conserved negatively charged region of RNF216, thus leading to a considerable change in the physicochemical and electrostatic profile of that domain that would inevitably affect its binding/interaction potential and would probably change its functional properties (e.g., loss of recognition and even interaction).

## Discussion

The present study investigated by high-throughput whole exome sequencing the genetic impact in patients with CHH. The seven patients of Cypriot origin with CHH/KS were identified with variants in genes linked with this phenotype: *ANOS1, SRA1, CHD7, WDR11, FGFR1, RNF216*, and *POLR3A*. A total of seven novel and two rare previously reported variants were identified in the patients of the current study and were found as novel or very rare in the ExAC population database (29). All these variants were also predicted to be pathogenic by at least two computational programs (22, 30–36). Our results once more confirmed the genetic complexity of CHH and the roles that exemplify a series of pleiotropic genes during development (13, 37, 38). More specifically, we identified the novel X-linked hemizygous truncated p.Gln82^*^ pathogenic variant in the *ANOS1* gene in a 28-year-old male with CHH (Table 1). Patients with sporadic KS/CHH due to *ANOS1* gene defects have been correlated with the phenotype of right renal agenesis/dysgenesis, thus provide evidence for the X-linked mode of inheritance and offering the opportunity for genetic counseling (12, 39–44). Approximately 10–20% of males with KS carry *ANOS1* variants or intragenic microdeletions (38). The majority of the X-linked KS variants cause alteration of splicing, frameshift or stop codons leading to synthesis of truncated anosmin (45). Nonsense variants and full deletions in the *ANOS1* gene are the most pathogenic and lead to a truncated and absent anosmin protein, respectively (37). Two of our normosmic male adult patients have been identified with the novel AD p.Pro186Ala and the previously reported p.Arg822Cys (46) variants in the *FGFR1* gene. Both of these patients were also characterized by delayed puberty during adolescence and later CHH. In a similar fashion with *ANOS1*, the expression of the *FGFR1* gene is also generated in the apparent olfactory bulbs and loss-of-function variants cause a form of KS with autosomal dominant inheritance (4–24, 28–50). FGFR1 is a cell surface membrane receptor that possesses tyrosine kinase activity and mediates fibroblast growth factor signaling (51). Patients with variants in the *FGFR1* gene present also various congenital anomalies that are not associated with the reproductive system and are often associated with kidney and tooth differentiation, ear and palate morphogenesis and the development of cortico-spinal axonal tracts (52). Notably, patient five, the 31-year-old male patient with CHH identified with the novel *FGFR1* p.Pro186Ala also shared in heterozygosity the novel *POLR3A* p.Arg561Gly missense variant. This finding adds to the already known spectrum of phenotypes resulting from *POLR3A* and *POLR3B* variants. *POLR3A* and *POLR3B* can be also associated to neurological or dental anomalies and isolated hypogonadotropic hypogonadism (53).

Patient 4 in addition to the novel AD *CHD7* p.Arg2400Trp variant also carried the known *MC4R* p.Val103Ile variant implicated in BMI and the novel p.Arg112gGln in the *PROP1* gene. Various studies have described *PROP1* gene variants as responsible for causing combined pituitary hormone deficiency (54–56). Heterozygous autosomal dominant loss-of-function variants in the *CHD7* gene are the major causal factor of CHARGE syndrome (57, 58), in addition to the fact that *CHD7* variants have been also been reported in patients with isolated CHH (59–61). Several reports have also linked *PROP1* variants with gonadotroph function that progressively declines and clinically patients with such variants may manifest a shortage of pubertal development, i.e., failure to enter or complete puberty (62, 63). There are several reports of spontaneous puberty with a posterior decline of gonadotrophic function that have been linked to p.Arg112Ter, p.Arg120Cys, p.Phe88Ser, and c.150delA *PROP1* variants (64–67). Since the *PROP1* gene is involved in the anterior pituitary, cell lineage specification variants could behave as an additive factor in the development of CHH when co-inherited with variants from genes involved in normal gonadotroph function. Such could be the case with the 18-year-old CHH patient of the present study identified with the novel *CHD7* p.Arg2400Trp and the novel p.Arg112gGln variant in the *PROP1* gene.

In the present study, a 72-year-old anosmic KS patient originally sought medical advice at the age of 40 in our clinic. Since then, he remains a patient of our clinic and at the age of 72-years he was identified with the novel AD p.Leu244Pro in the *WDR11* gene. WDR11 has been implicated in CHH and KS, human developmental genetic disorders defined by delayed puberty and infertility (68, 69). Several reports in CHH patients with and without anosmia identified in heterozygosity variants in the *WDR11* gene (68). *WDR11* is expressed in several adult organs including the brain and the gonads. Comprehensive analysis of the mouse brain displayed *WDR11* expression in the GnRH neuronal migratory location including nasal cavity and cribriform plate area in E12.5 mouse embryo as well as the median eminence in the adult brain, showing co-localization with GnRH. Furthermore, *WDR11* is expressed all over the developing and adult olfactory bulb (OB) and its WD domains are important for β-propeller formation and protein-protein interaction (70). In addition, *WDR11* interacts with *EMX1*, a homeodomain transcription factor involved in the development of olfactory neurons, and missense variants diminish or eliminate this interaction (68). Therefore, it is highly likely that the impaired pubertal development in these patients results from a deficiency of productive WDR11 protein interaction.

Interestingly, two out of the seven CHH patients in our cohort, a 30-year-old female (Patient 7) and a 19-year-old (Patient 3) male were both identified with variants in the *SRA1* gene. More specifically, the 30-year-old female carried in homozygosity the previously reported p.Ile179Thr variant in the *SRA1* gene (25). The 19-year-old male also carried this same *SRA1* variant in heterozygosity together with the novel p.Asp792Asn variant in the *RNF216* gene. As reported by Kotan et al. (25), the variant p.Ile179Thr was reported only once in one independent Turkish family with IHH/delayed puberty and its severity was supported by functional studies. Using a mutant *SRA1* construct, reduced co-activation of ligand-dependent activity of the estrogen receptor alpha was demonstrated (25).

The variant p.Ile179Thr was not found in 51 Cypriots used as controls for the purposes of the present study and was reported with an allele frequency of 0.00081 in GnomAD v2.1.1. Therefore, most likely, a hot spot exists for this specific variant in the greater Eastern Mediterranean region, suggesting a founder effect phenomenon, which has been also seen for other rare endocrine conditions in this area (71).

The *SRA1* is a steroid receptor RNA activator that has been shown to positively regulate the activity of the androgen receptor and the estrogen receptor (72, 73). In recent years only a few studies linked the *SRA1* gene as responsible for causing CHH when patients inherit pathogenic variants in the AR form (25, 26).

The concepts of incomplete penetrance and variable expressivity have been notified in such cases as the 19-year-old patient where digenic variants are observed. Digenic variants account for variable phenotypes in idiopathic hypogonadotropic hypogonadism and other disorders and several recent and older reports identified such conditions (17, 40, 74, 75). The existence of digenic and oligogenic inheritance in CHH is quite common, with about 20% of CHH cases reported to share at least two causative variants that could result in a disease phenotype (1, 15). The most appropriate way to examine the possibility of low penetrance and variable expressivity of CHH genes is by concurrently carrying out targeted genetic analyses, or preferably by performing WES on the probands and available relatives so as to establish digenic or oligogenic transmission.

## Review of the Literature

Congenital hypogonadotropic hypogonadism (CHH) is a rare disorder of sexual maturation characterized by GnRH deficiency with low sex steroid levels associated with low levels of LH and FSH. CHH may be caused by variants in numerous genes and recent studies shed light on the complexity of CHH genetics (6, 15, 76). Over the recent few years, genetic evaluation of patients with inherited diseases, including CHH, has increasingly utilized massive parallel sequencing by next-generation sequencing (NGS), that allows the concurrent investigation of thousands of genes (1, 15, 77). At this scale of analysis, NGS is inexpensive and rapid compared to the traditional Sanger sequencing and is increasingly being used in medical practice. NGS has certainly facilitated CHH genetic diagnoses and aided healthcare professionals to provide reliable and informed genetic counseling for patients with CHH. The crucial challenge regarding NGS concerns the identification of true oligogenism in circumstances involving several rare variants which do not have a clear phenotypic effect and are identified by coincidence. Such a challenge also concerns the identification of genes underlying CHH pathogenesis and which are likewise reported to act in an oligogenic context (78). Since the discovery of *ANOS1* (79), more than sixty genes have been reported to underlie CHH and were previously considered to be inherited in the AD form (6). Herein, we review six of these genes: *ANOS1, FGFR1, CHD7, WDR11, RNF216*, and *POLR3A*, since novel variants in these genes have been identified in our cohort of patients under investigation.

### *ANOS1 (KAL1)* Gene Variants Causing X-Linked Recessive KS/CHH

*ANOS1* was the first gene linked to Kallmann syndrome (KS) and since the early nineties when the first reports demonstrated variants with an X-linked mode of inheritance (80–83), many others followed throughout the years (6, 84–88). KS occurs more frequently in males than in females, with an estimated prevalence of 1 in 30,000 males and 1 in 120,000 females (12). Patients with KS associated with *ANOS1* pathogenic variants usually exhibit anosmia accompanied with CHH (12, 14, 85, 86). Fewer patients with pathogenic variants in *ANOS1* are either anosmic or hyposmic and have been reported to exhibit other signs, such as mirror movements and renal agenesis, but they do not always co-segregate with the variant recognized in a given family (85, 89–91). According to the *Human Gene Mutation Database* (http://www.hgmd.cf.ac.uk/ac/index.php) more than 150 *ANOS1* pathogenic variants have been reported as the causative factor in KS patients. Most of these pathogenic variants mainly consist of nucleotide deletions or insertions and to a lesser extend of variants that involve amino acid missense substitutions (88, 92, 93). The *ANOS1* gene encodes anosmin, a protein which plays a significant role in the embryogenesis of brain, kidneys, respiratory and digestive systems (92, 94). Anosmin, as an extracellular matrix protein binds to the cell membrane and stimulates the development of the olfactory system and behaves as an axonal guidance for the GnRH neurons, the olfactory cells and the Purkinje cerebellum neurons (95). Monogenic loss-of-function pathogenic variants in *ANOS1* gene have been estimated to account for 4–10% of KS/CHH cases and has been principally studied in many reported cohorts (12, 41–44, 88, 96–99).

Regarding the reproductive phenotype, male KS patients with *ANOS1* variants display a complete penetrance of CHH and their pre- and postnatal gonadotropin deficit is severe with a high frequency of micropenis, cryptorchidism and complete absence of gonadal development (15, 16).

### *FGFR1* Gene Variants Causing KS/CHH

The presence of variants in the *FGFR1* gene is another important cause of KS and was the first gene to be identified as an AD form of the disease (49, 100). More than 140 loss-of-function mutations in the *FGFR1* gene have been reported with missense, non-sense and frameshift defects being the most frequent (101). Less frequent autosomal gene deletions have also been reported in patients with CHH and KS (102, 103). *FGFR1* is considered to be a pleiotropic gene that can display different roles during development and variants found in it can cause CHH with or without anosmia (49, 100). Genotype-phenotype correlations in patients with AD variants in the *FGFR1* gene demonstrated some clinical features linked with KS, such as loss of nasal cartilage, hearing deficit and anomalies of the limbs (6, 93, 101). The function of *FGFR1* in the normal development of the olfactory bulb proposes the link of anosmia with GnRH deficiency in the *FGFR1*-mutated patients (104). Phenotype analysis proposes that *FGFR1* elaborates in the normal migration of GnRH fetal neurons, but this is not entirely clear-cut as a considerable proportion of *FGFR1*-mutated patients have normosmic GnRH deficiency (15). Regarding the reproductive phenotype of male patients with *FGFR1* variants, the penetrance of CHH and GnRH deficiency is variable and ranges from profound to partial puberty and even to reversal (1, 99, 105, 106).

Several groups have reported patients harboring *FGFR1* variants linked to non-reproductive signs. Patients with *FGFR1* mutations have been reported to suffer from health conditions such as 8p11 myeloproliferative syndrome (107, 108), encephalocraniocutaneous lipomatosis (109, 110), Hartsfield syndrome, a rare condition characterized by holoprosencephaly, which is an abnormality of brain development (111, 112), osteoglophonic dysplasia, a condition characterized by abnormal bone growth that leads to craniofacial abnormalities and dwarfism (113, 114) and Pfeiffer syndrome, which is characterized by craniosynostosis (115, 116). Somatic pathogenic variants involving the *FGFR1* gene have also been reported in several types of cancers, including the lung, breast, esophagous, oral cavity and brain tumors (101, 117–119). Taking into consideration the genotypic and phenotypic heterogeneity that is observed in patients with *FGFR1* variants and the fact that their prevalence is not clearly established makes genetic counseling rather complicated.

### *CHD7* Gene Variants Causing CHARGE Syndrome and CHH/KS

*CHD7* is the gene that codes for the chromodomain helicase DNA binding protein 7 and variants found in the AD form were first reported as the genetic cause in a series of patients with CHARGE (coloboma, heart defect, atresia choanae, growth retardation, genital abnormality, and ear abnormality) syndrome (57, 120). CHARGE syndrome occurs in approximately 1 in 8,500 to 10,000 new-borns and up-to-date more than 600 *CHD7* variants in the AD form have been associated with the disorder (57, 121–124). Several other studies of families carrying *CHD7* mutations in the AD form also demonstrated a broad phenotypic variability and linked more than 50 of them with KS and congenital hypogonadotropic hypogonadism (59, 60, 69, 125). It has been estimated that inherited and *de novo CHD7* mutations account for 5–10 percent of all cases of KS and an accountable number of these patients exhibit mild form features of CHARGE syndrome, such as abnormally shaped ears, hearing loss, hare lip/cleft palate and cardiac abnormalities (60, 61, 69, 121, 126).

### *WDR11* Gene Variants Causing CHH/KS

WDR11 is a member of the WD-repeat containing protein family and comprises of twelve conserved domains of approximately 40 amino acids (68). The *WDR11* gene is located in the chromosome 10q25-26 region and is expressed in various human organs including the brain, ear, lung, heart, kidney and the gonads (70). WDR11 is a scaffolding protein that is involved in multiple of cellular proceedings, including cell cycle progression, signal transduction, apoptosis and gene regulation (70). Kim et al. first reported that when mutated *WDR11* is linked with idiopathic HH and KS (68). Since the initial report by Kim et al. (68), a few others followed and linked the *WDR11* gene with different pathogenic variants in male patients without anosmia and CHH (127, 128). Recently, the *WDR11* gene has also been shown to be involved in the Hedgehog (Hh) signaling pathway which is important for the normal ciliogenesis and when mutated can be the causal factor of KS and HH (68, 70). Another recent report by Sutani et al. (129) linked *WDR11* as another causative gene for coloboma, cardiac anomaly and growth retardation in the *10q26* deletion syndrome.

### *RNF216* Gene Variants Linked to Gordon Holmes Syndrome

The RNF216 protein is a cytoplasmic protein which interacts with the serine/threonine protein kinase i.e., the receptor-interacting protein (RIP). Particular zinc finger domains of the RNF216 protein are necessary for its interaction with RIP and for the inhibition of TNF- and IL1-induced NF-kappa B activation pathways (130, 131). Additionally, the RNF216 protein plays a role in the ubiquitin-proteasome system for the break-down and degradation of unwanted proteins. Specifically, this protein functions as an E3 ubiquitin ligase (132). Variants in the *RNF216* gene have been linked with hypogonadotropic hypogonadism, ataxia and dementia (28). More explicitly digenic homozygous variants in *RNF216* and *OTUD4*, which encode a ubiquitin E3 ligase and a deubiquitinase, respectively, were identified in three affected siblings in a consanguineous family (28). Several other recent studies also reported variants in the *RNF216* gene as a result of consanguinity to cause Gordon Holmes syndrome, a rare disorder characterized by diminished production of hormones leading to hypogonadotropic hypogonadism and difficulty in the coordination of movements i.e., cerebellar ataxia (133–136). These recent findings regarding the *RNF216* gene associate the disorderly ubiquitination to neurodegeneration and reproductive dysfunction in combination with functional studies to reveal specific genetic interactions that cause disease.

### *POLR3A* Gene Variants Associated With Hypomyelinating Leukodystrophy and HH

The *POLR3A* gene provides instructions for the production of the largest subunit of RNA polymerase III which is the enzyme involved in the RNA synthesis (137). The gene is located in chromosome *10q22.3* and variants inherited in the AR form have been initially reported in French-Canadian families with hypomyelinating leukodystrophy (138). Interestingly, these families were mapped to the same locus as leukodystrophy with oligodontia and demonstrated clinical and radiological overlap with patients with hypomyelination, hypodontia and hypogonadotropic hypogonadism syndrome (138). Several other recent studies that followed also reported variants in the *POLR3A* gene as being responsible for causing hypomyelination, hypodontia and hypogonadotropic hypogonadism, thus establishing a series of *POLR3A* gene variants to be associated with polymerase III-related leukodystrophy (139–144). It is estimated that 30–40% of patients with leukodystrophies remain without a molecular diagnosis (138, 141). The existence of mild and overlapping hypomyelinating leukodystrophy phenotypes could be attributed to heterozygous variants found in the *POLR3A* gene as a result of an abnormal enzymatic function of the RNA Pol III catalytic subunit. The role of heterozygosity in *POLR3A* in the overall pathogenesis of CHH is not well-established, and the possibility of a synergistic effect between these variants and variants identified in other genes cannot be excluded. Additionally, *POLR3A* gene could also be speculated to be a *phenocopy* gene due to the observed variability of phenotypes, therefore, patients and family members identified with mutations in this gene should be re-evaluated for understated and previously unrecognized clinical signs.

## Conclusion

GnRH deficiency has been recognized both clinically and genetically as a heterogeneous disease with a range of different reproductive phenotypes including of congenital GnRH deficiency with anosmia (KS) and congenital GnRH deficiency with normal olfaction (normosmic CHH). The present study/review discusses the involvement of known and novel variants in patients with CHH/KS and adds up to the ontogeny of GnRH deficiency.

Moreover, this study provides new genetic findings and reinforces the significance of the use of NGS technology for the accurate molecular diagnosis and treatment of this rare condition.

## Data Availability Statement

The datasets from this study can be found in DRYAD repository (https://datadryad.org) under accession number doi: 10.5061/dryad.1vhhmgqqj Link: https://datadryad.org/stash/share/uhb7bIQj8JDLq9TKTtb51pF7W1xprCFi58lt7vcRxU4.

## Ethics Statement

The studies involving human participants were reviewed and approved by The Cyprus National Ethics Committee. Written informed consent to participate in this study was provided by the participants' legal guardian/next of kin. Written, informed consent was obtained from all seven adult individuals that participate in the study for the publication of any potentially identifiable images or data included in this article.

## Author Contributions

VN, PF, MT, NS, and LP contributed to the conception, design, and interpretation of the project. MS, FC, AO, GS, and DV contributed to the experimental part and data analysis of the project. NN, GT, and CM contributed to drafting or revising intellectual content of the manuscript. VN, NS, and LP had primary responsibility for final content. All authors read and approved the final manuscript.

## Conflict of Interest

The authors declare that the research was conducted in the absence of any commercial or financial relationships that could be construed as a potential conflict of interest.

## Supplementary Material

Supplementary Figure 1 Sequence electropherograms of the novel *SRA1* p.Ile179Thr variant in heterozygous and homozygous state. The variant is indicated by a red arrow.

Supplementary Figure 2 Sequence electropherograms of the **(A)** p.Pro186Ala and **(B)** p.Arg822Cys *FGFR1* variants in heterozygous state. The variants are indicated by red arrows.

Supplementary Figure 3 Sequence electropherogram of the *POLR3A* p.Arg561Gly variant in heterozygous state. The variant is indicated by a red arrow.

Supplementary Figure 4 Sequence electropherogram of the novel *PROP1* p.Arg112Gln variant in heterozygous state. The variant is indicated by a red arrow.

Supplementary Figure 5 Family pedigrees and inheritance patterns of the **(A)** *ANOS1:*p.Gln82^*^ mutation in patient 1 and the **(B)** *SRA1*:p.Ile179Thr, *RNF216*:p.Asp792Asn variations in patient 3.

Supplementary Figure 6 Predicted pathogenicity of the due to digenicity by ORVAL platform. **(A)** S-plot and corresponding table with score values of the results for the *FGFR1/POLR3A* digenic combination. **(B)** S-plot and corresponding table with score values of the results for the *RNF216/SRA1* digenic combination. Black arrows indicate the disease-causing position of the digenic combination on the plot. Y-axis, Support Score: percentage of individual predictors agreeing on the disease-causing class of the digenic combination. X-axis, Classification Score: median probability among all predictors that the digenic combination is disease-causing. The higher the scores, the more confident the predictor is for the disease-causing class.

Supplementary Table 1 Genes with biological involvement in the GnRH neuronal system and CHH selected for variant analysis of the WES. For each gene the corresponding genetic and phenotypic information is indicated. The mean target coverage of the target region for each gene is designated in a separate column.
