# Supplementary figures and images for: GnRH Deficient Patients With Congenital Hypogonadotropic Hypogonadism: Novel Genetic Findings in ANOS1, RNF216, WDR11, FGFR1, CHD7, and POLR3A Genes in a Case Series and Review of the Literature
Source: Front Endocrinol (Lausanne). 2020 Aug 28;11:626. doi: 10.3389/fendo.2020.00626 (PMC7485345; doi:10.3389/fendo.2020.00626)

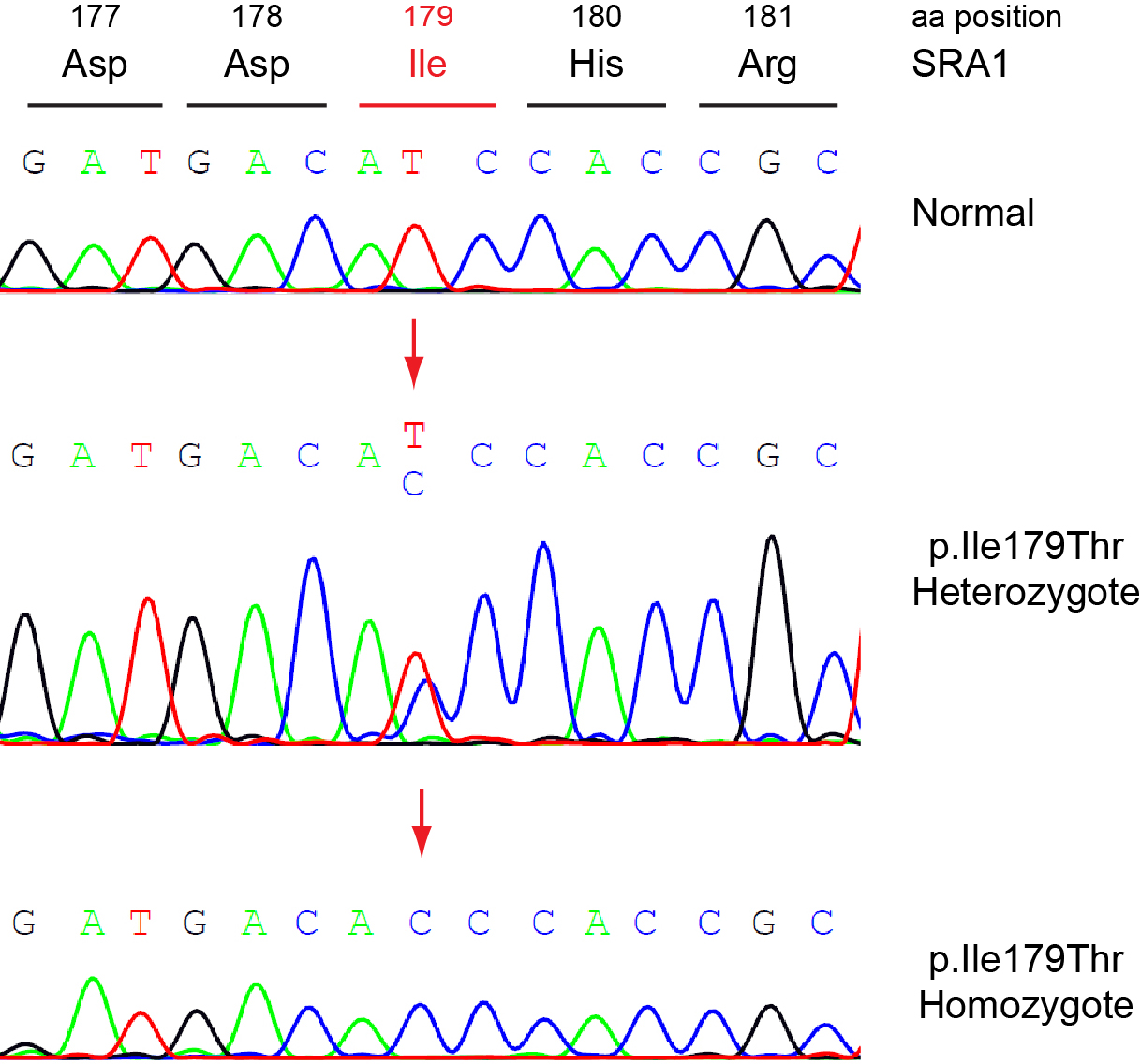

Supplement: Supplementary Figure 1 — Sequence electropherograms of the novel SRA1 p.Ile179Thr variant in heterozygous and homozygous state. The variant is indicated by a red arrow. [file Image_1.JPEG]

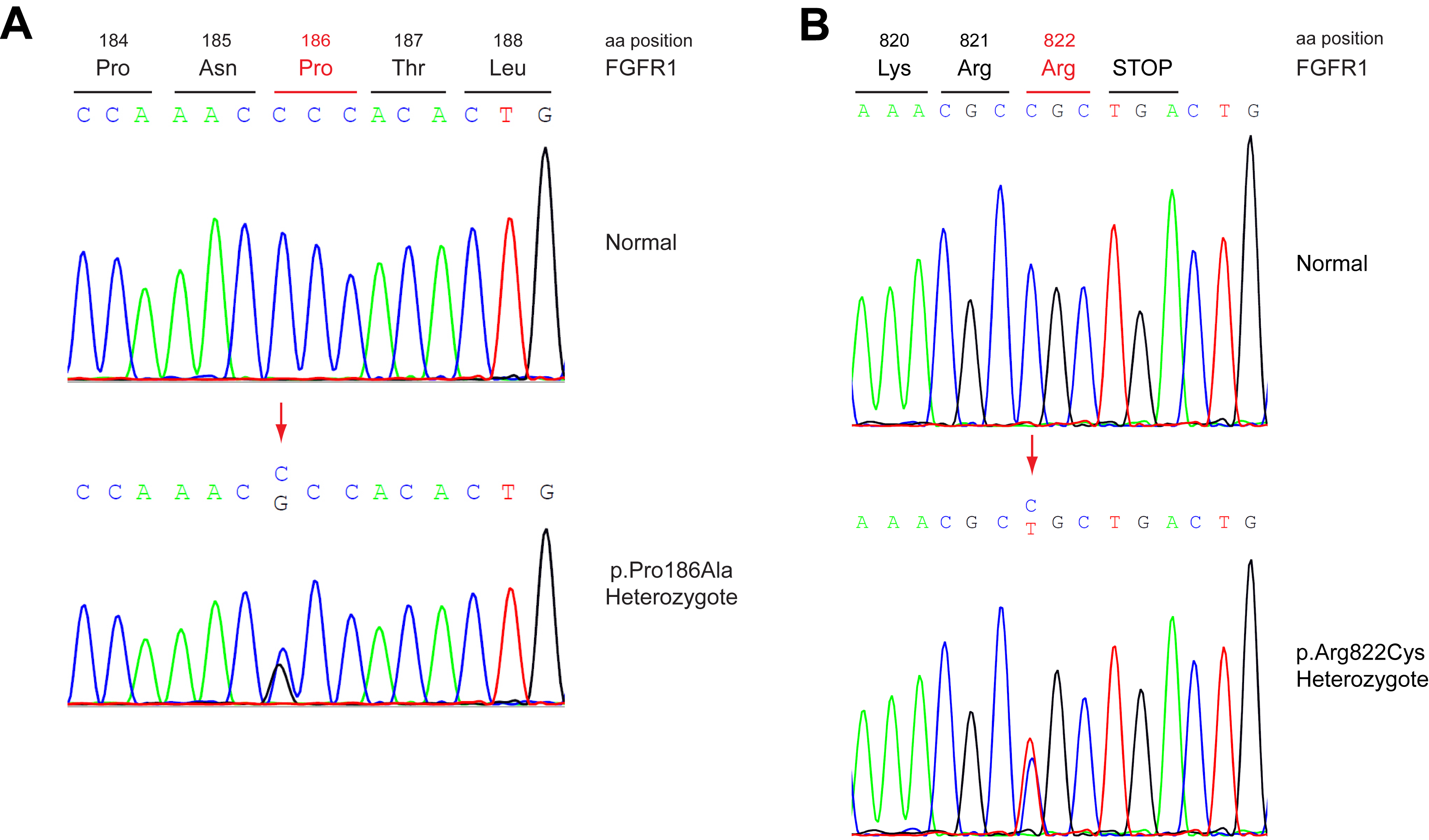

Supplement: Supplementary Figure 2 — Sequence electropherograms of the (A) p.Pro186Ala and (B) p.Arg822Cys FGFR1 variants in heterozygous state. The variants are indicated by red arrows. [file Image_2.JPEG]

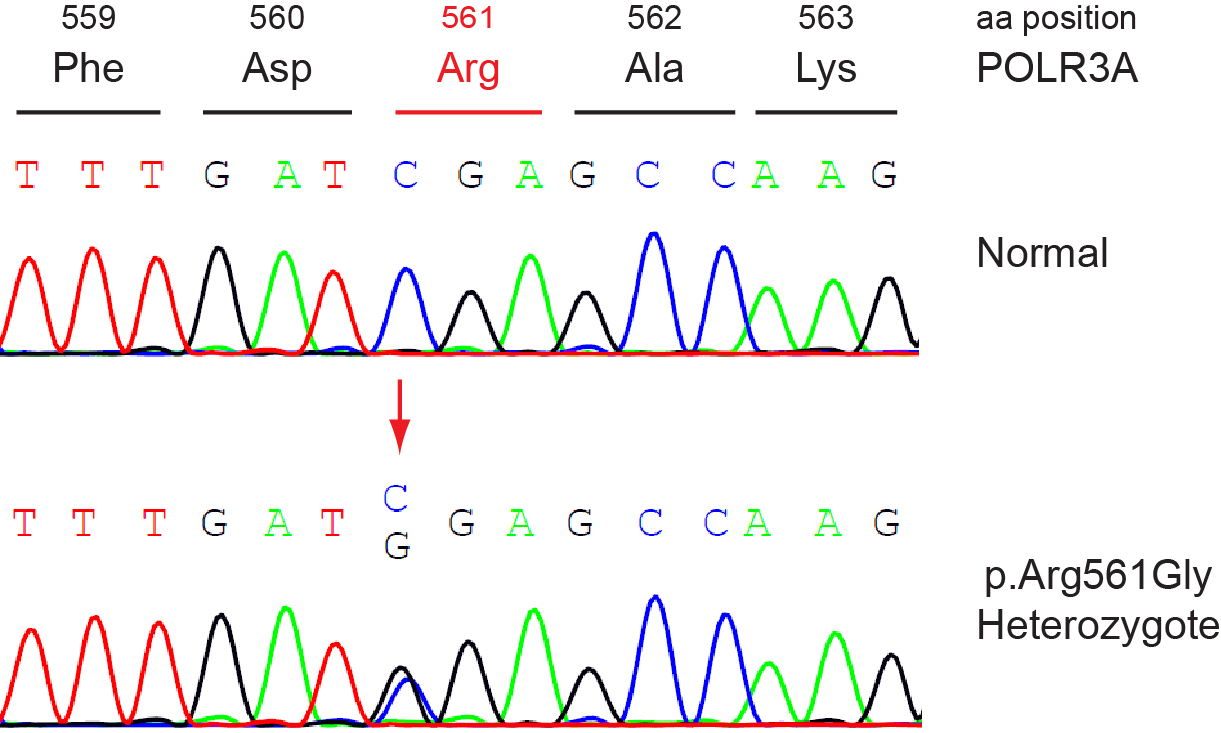

Supplement: Supplementary Figure 3 — Sequence electropherogram of the POLR3A p.Arg561Gly variant in heterozygous state. The variant is indicated by a red arrow. [file Image_3.JPEG]

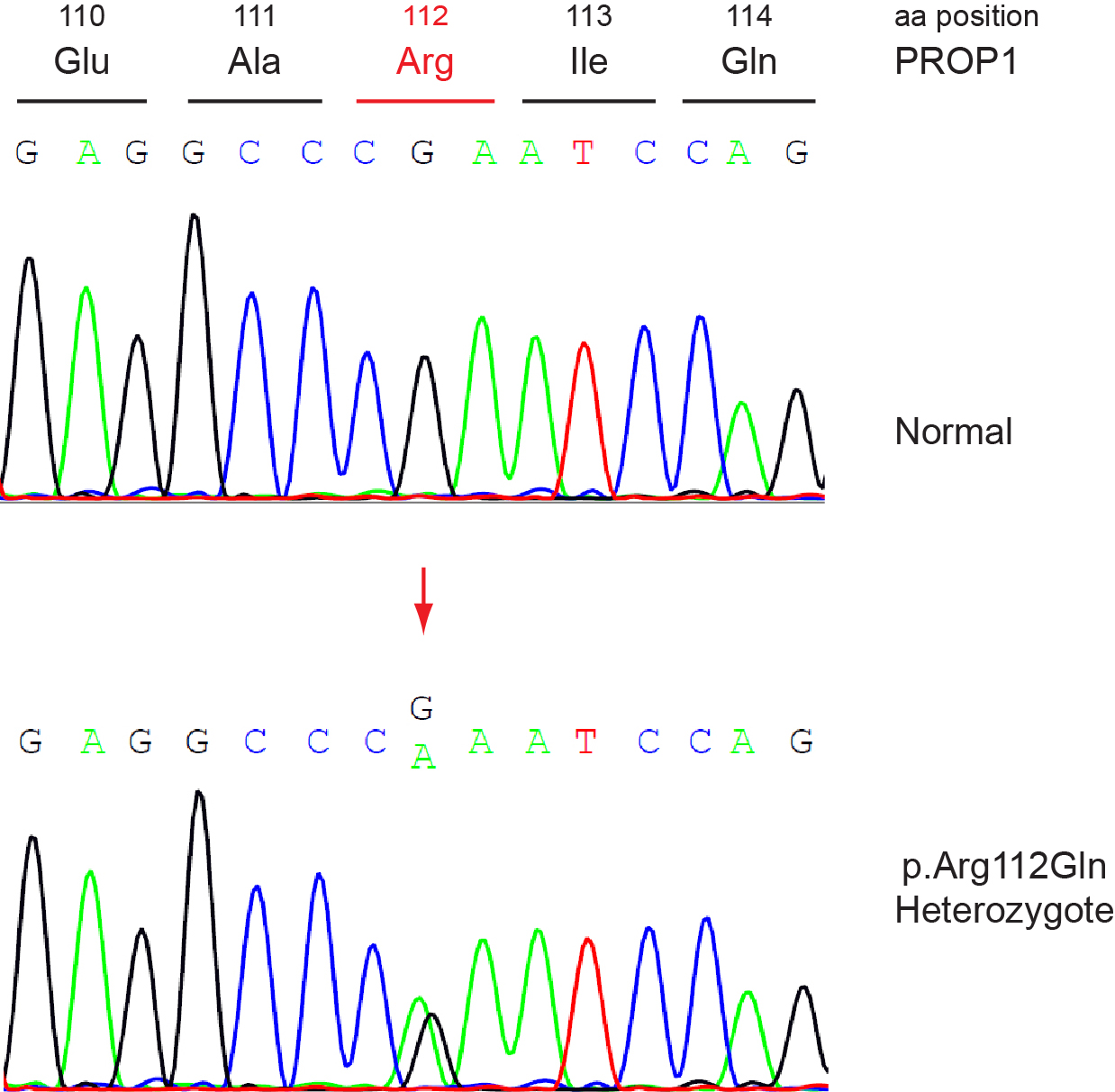

Supplement: Supplementary Figure 4 — Sequence electropherogram of the novel PROP1 p.Arg112Gln variant in heterozygous state. The variant is indicated by a red arrow. [file Image_4.JPEG]

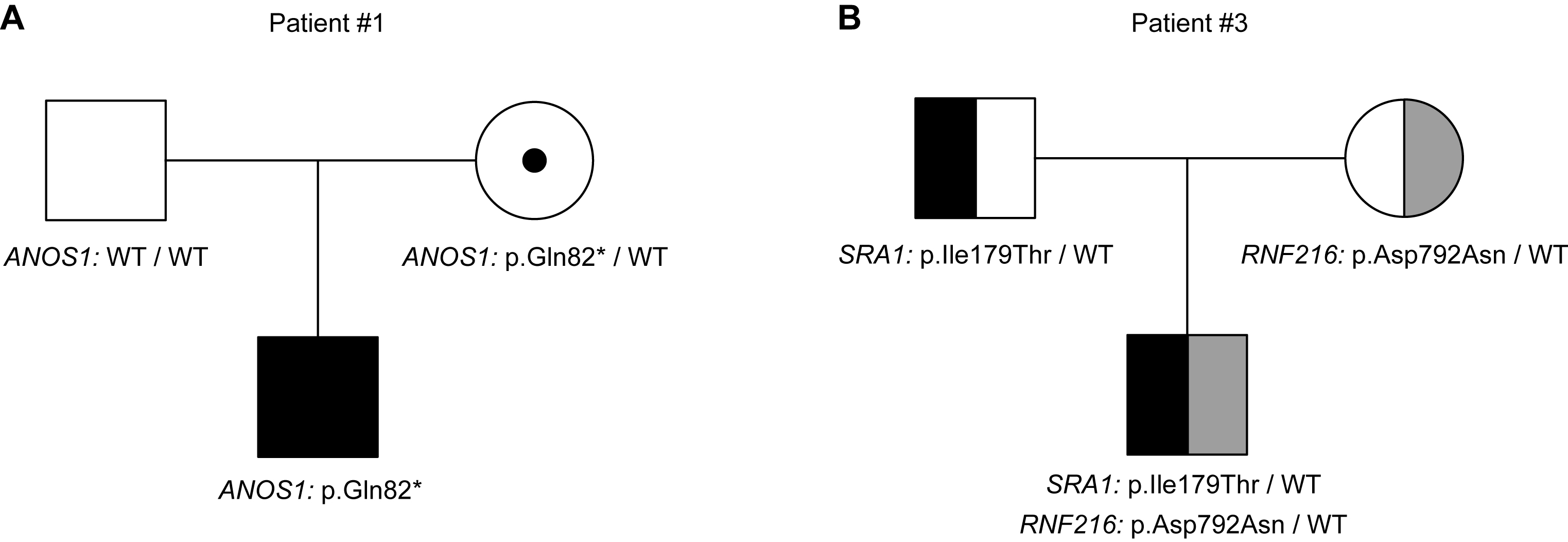

Supplement: Supplementary Figure 5 — Family pedigrees and inheritance patterns of the (A) ANOS1:p.Gln82* mutation in patient 1 and the (B) SRA1:p.Ile179Thr, RNF216:p.Asp792Asn variations in patient 3. [file Image_5.JPEG]

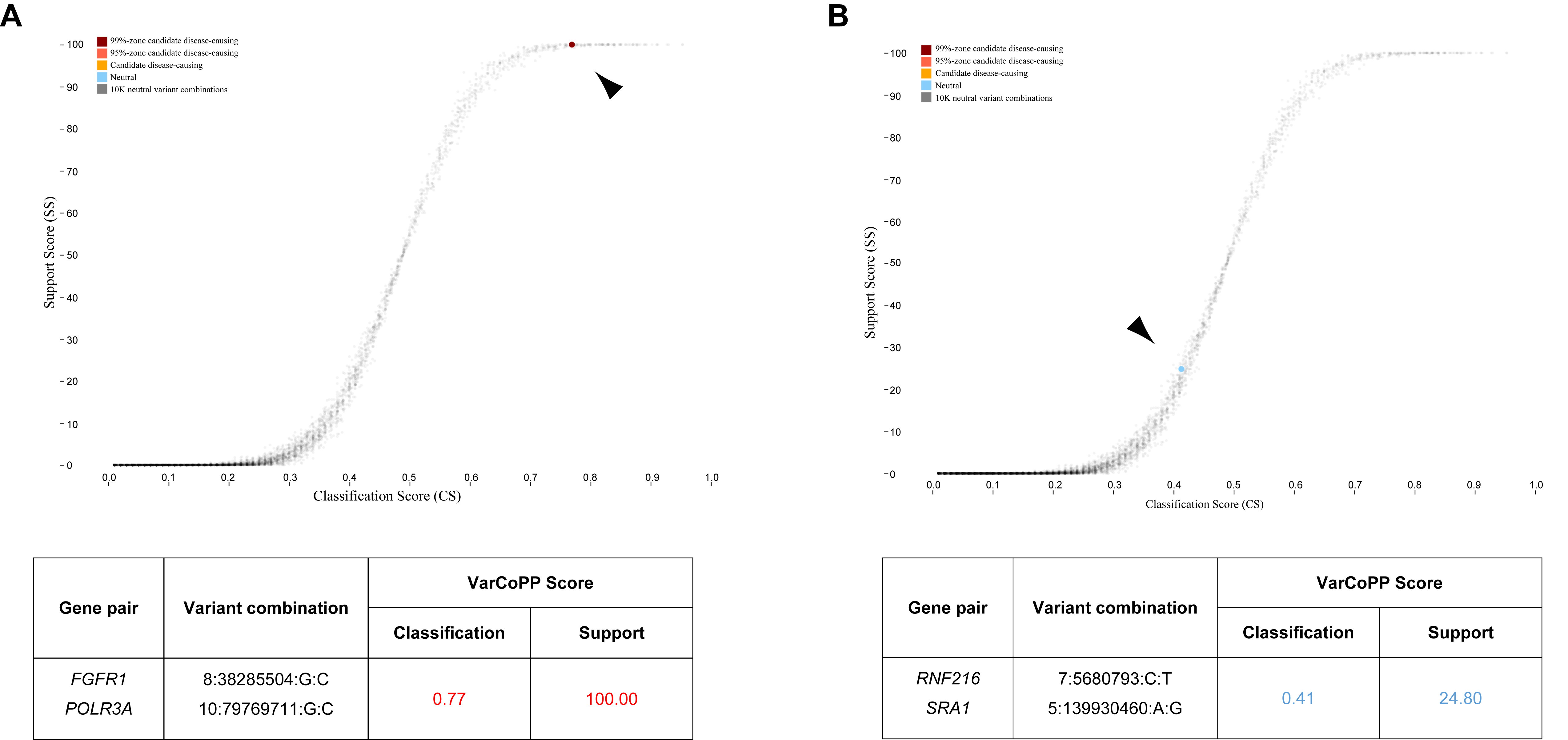

Supplement: Supplementary Figure 6 — Predicted pathogenicity of the due to digenicity by ORVAL platform. (A) S-plot and corresponding table with score values of the results for the FGFR1/POLR3A digenic combination. (B) S-plot and corresponding table with score values of the results for the RNF216/SRA1 digenic combination. Black arrows indicate the disease-causing position of the digenic combination on the plot. Y-axis, Support Score: percentage of individual predictors agreeing on the disease-causing class of the digenic combination. X-axis, Classification Score: median probability among all predictors that the digenic combination is disease-causing. The higher the scores, the more confident the predictor is for the disease-causing class. [file Image_6.JPEG]
