# Supplementary material for: GnRH Deficient Patients With Congenital Hypogonadotropic Hypogonadism: Novel Genetic Findings in ANOS1, RNF216, WDR11, FGFR1, CHD7, and POLR3A Genes in a Case Series and Review of the Literature
Source: Front Endocrinol (Lausanne). 2020 Aug 28;11:626. doi: 10.3389/fendo.2020.00626 (PMC7485345; doi:10.3389/fendo.2020.00626)
Supplement: Supplementary Table 1 — Genes with biological involvement in the GnRH neuronal system and CHH selected for variant analysis of the WES. For each gene the corresponding genetic and phenotypic information is indicated. The mean target coverage of the target region for each gene is designated in a separate column. [file Table_1.DOCX]

**Supplementary Table 1.** **Genes with biological involvement in the GnRH neuronal system and CHH selected for variant analysis of the WES.** For each gene the corresponding genetic and phenotypic information is indicated. The mean target coverage of the target region for each gene is designated in a separate column.

| **Gene** | **Phenotype** | **Transcript ID** | **Reference Sequence (RefSeq)** | **OMIM ID** | **Reference** | **Mean target coverage** |
| --- | --- | --- | --- | --- | --- | --- |
| *ANOS1* | CHH | ENST00000262648.8 | NM_000216 | 300836 | ([1](#_ENREF_1)) | 30.73X |
| *CHD7* | CHH | ENST00000423902.7 | NM_017780 | 608892 | ([2](#_ENREF_2)) | 61.84X |
| *DLK1* | GnRH neuronal system (PP) | ENST00000341267.9 | NM_001317172 | 176290 | ([3](#_ENREF_3)) | 15.21X |
| *DMXL2* | GnRH neuronal system (CHH) | ENST00000543779.6 | NM_015263 | 612186 | ([4](#_ENREF_4)) | 63.24X |
| *DUSP6* | CHH | ENST00000279488.8 | NM_001946 | 602748 | ([5](#_ENREF_5)) | 24.8X |
| *IRF2BPL (EAP1)* | GnRH neuronal system | ENST00000238647.5 | NM_024496 | 611720 | ([6](#_ENREF_6)) | 24.94X |
| *EBF2* | GnRH neuronal system | ENST00000520164.6 | NM_022659 | 609934 | ([7](#_ENREF_7)) | 23.26X |
| *FEZF1* | CHH | ENST00000442488.7 | NM_001024613 | 613301 | ([8](#_ENREF_8)) | 49.94X |
| *FGF17* | CHH | ENST00000359441.4 | NM_003867 | 603725 | ([5](#_ENREF_5)) | 44.22X |
| *FGF8* | CHH | ENST00000320185.6 | NM_006119 | 600483 | ([9](#_ENREF_9)) | 78.69X |
| *FGFR1* | CHH | ENST00000447712.7 | NM_001174063 | 136350 | ([10](#_ENREF_10)) | 59.3X |
| *FLRT3* | CHH | ENST00000341420.5 | NM_013281 | 604808 | ([5](#_ENREF_5)) | 39.35X |
| *FSHB* | CHH | ENST00000417547.1 | NM_000510 | 136530 | ([11](#_ENREF_11)) | 42.53X |
| *GHSR* | GnRH neuronal system | ENST00000241256.3 | NM_004122 | 601898 | ([12](#_ENREF_12)) | 105.79X |
| *GNRH1* | CHH | ENST00000421054.7 | NM_001083111 | 152760 | ([13](#_ENREF_13)) | 82.74X |
| *GNRHR* | CHH | ENST00000226413.5 | NM_000406 | 138850 | ([14](#_ENREF_14)) | 33.49X |
| *HESX1* | GnRH neuronal system (PHD) | ENST00000295934.8 | NM_001376058 | 601802 | ([15](#_ENREF_15)) | 70.94X |
| *HS6ST1* | CHH | ENST00000259241.7 | NM_004807 | 604846 | ([16](#_ENREF_16)) | 41.68X |
| *IGFALS* | GnRH neuronal system | ENST00000415638.3 | NM_001146006 | 601489 | ([17](#_ENREF_17)) | 103.36X |
| *IGSF1* | GnRH neuronal system | ENST00000370903.8 | NM_001170961 | 300137 | ([18](#_ENREF_18)) | 36.45X |
| *IGSF10* | GnRH neuronal system | ENST00000282466.3 | NM_178822 | 617351 | ([19](#_ENREF_19)) | 68.73X |
| *IL17RD* | CHH | ENST00000296318.12 | NM_017563 | 606807 | ([5](#_ENREF_5)) | 18.63X |
| *KISS1* | CHH | ENST00000367194.5 | NM_002256 | 603286 | ([20](#_ENREF_20)) | 81.19X |
| *KISS1R* | CHH | ENST00000234371.10 | NM_032551 | 604161 | ([21](#_ENREF_21)) | 54.73X |
| *LEP* | GnRH neuronal system | ENST00000308868.5 | NM_000230 | 164160 | ([22](#_ENREF_22)) | 22.39X |
| *LEPR* | GnRH neuronal system | ENST00000349533.11 | NM_002303 | 601007 | ([23](#_ENREF_23)) | 52.12X |
| *LHB* | CHH | ENST00000649238.3 | NM_000894 | 152780 | ([24](#_ENREF_24)) | 114.06X |
| *LHX3* | GnRH neuronal system (PHD) | ENST00000371746.9 | NM_001363746 | 600577 | ([25](#_ENREF_25)) | 36.09X |
| *LIN28B* | GnRH neuronal system (PP) | ENST00000637759.1 | NM_001004317 | 611044 | ([26](#_ENREF_26)) | 10.92X |
| *MC4R* | GnRH neuronal system | ENST00000299766.5 | NM_005912 | 155541 | ([27](#_ENREF_27), [28](#_ENREF_28)) | 84.48X |
| *MKRN3* | GnRH neuronal system (PP) | ENST00000314520.6 | NM_005664 | 603856 | ([29](#_ENREF_29)) | 57.54X |
| *MSX1* | GnRH neuronal system | ENST00000382723.5 | NM_002448 | 142983 | ([30](#_ENREF_30)) | 31.01X |
| *NR0B1* | GnRH neuronal system | ENST00000378970.5 | NM_000475 | 300473 | ([31](#_ENREF_31)) | 47.87X |
| *NR5A1* | GnRH neuronal system | ENST00000373588.9 | NM_004959 | 184757 | ([32](#_ENREF_32)) | 42.4X |
| *NSMF* | CHH | ENST00000371475.9 | NM_015537 | 608137 | ([33](#_ENREF_33)) | 61.05X |
| *OTUD4* | GnRH neuronal system | ENST00000447906.8 | NM_017493 | 611744 | ([34](#_ENREF_34)) | 50.82X |
| *PCSK1* | GnRH neuronal system | ENST00000311106.8 | NM_000439 | 162150 | ([35](#_ENREF_35)) | 73.04X |
| *PNPLA6* | GnRH neuronal system | ENST00000414982.7 | NM_006702 | 603197 | ([36](#_ENREF_36), [37](#_ENREF_37)) | 111.62X |
| *POLR3A* | GnRH neuronal system | ENST00000372371.8 | NM_007055 | 614258 | ([38](#_ENREF_38), [39](#_ENREF_39)) | 122.24X |
| *POLR3B* | GnRH neuronal system | ENST00000228347.9 | NM_018082 | 614366 | ([38](#_ENREF_38), [40](#_ENREF_40)) | 130.05X |
| *POU1F1* | GnRH neuronal system (PHD) | ENST00000344265.7 | NM_000306 | 173110 | ([41](#_ENREF_41)) | 82.24X |
| *PROK2* | CHH | ENST00000295619.4 | NM_001126128 | 607002 | ([42](#_ENREF_42)) | 34.03X |
| *PROKR2* | CHH | ENST00000217270.3 | NM_144773 | 607123 | ([42](#_ENREF_42)) | 75.11X |
| *PROP1* | GnRH neuronal system (PHD) | ENST00000308304.2 | NM_006261 | 601538 | ([43](#_ENREF_43)) | 46.91X |
| *RNF216* | CHH | ENST00000389902.7 | NM_207111 | 609948 | ([34](#_ENREF_34)) | 39.97X |
| *SEMA3A* | CHH | ENST00000265362.9 | NM_006080 | 603961 | ([44](#_ENREF_44)) | 25.8X |
| *SEMA3E* | GnRH neuronal system | ENST00000643230.2 | NM_012431 | 608166 | ([45](#_ENREF_45)) | 57.03X |
| *SEMA7A* | GnRH neuronal system | ENST00000261918.9 | NM_003612 | 607961 | ([46](#_ENREF_46)) | 34.8X |
| *SOX10* | CHH | ENST00000396884.8 | NM_006941 | 602229 | ([47](#_ENREF_47)) | 32.27X |
| *SOX2* | GnRH neuronal system | ENST00000325404.3 | NM_003106 | 184429 | ([48](#_ENREF_48)) | 54.42X |
| *SOX3* | GnRH neuronal system | ENST00000370536.4 | NM_005634 | 313430 | ([49](#_ENREF_49)) | 28.01X |
| *SPRY4* | CHH | ENST00000344120.4 | NM_001127496 | 607984 | ([5](#_ENREF_5)) | 10.06X |
| *SRA1* | GnRH neuronal system (CHH) | ENST00000336283.7 | NM_001035235 | 603819 | ([50](#_ENREF_50)) | 32.38X |
| *SRY* | Gonadal system | ENST00000383070.2 | NM_003140 | 480000 | ([51](#_ENREF_51)) | 92.16X |
| *STUB1* | GnRH neuronal system | ENST00000219548.9 | NM_005861 | 607207 | ([52](#_ENREF_52)) | 103.36X |
| *TAC3* | CHH | ENST00000393867.5 | NM_013251 | 162330 | ([53](#_ENREF_53)) | 101.76X |
| *TACR1* | GnRH neuronal system | ENST00000305249.10 | NM_001058 | 162323 | ([54](#_ENREF_54)) | 23.41X |
| *TACR3* | CHH | ENST00000304883.3 | NM_001059 | 162332 | ([53](#_ENREF_53)) | 41.06X |
| *WDR11* | CHH | ENST00000263461.11 | NM_018117 | 606417 | ([55](#_ENREF_55)) | 134.08X |

PP, Precocious Puberty; WES, Whole Exome Sequencing; PHD, Pituitary Hormone Deficiency

**References**

1. Hardelin JP, Levilliers J, Blanchard S, Carel JC, Leutenegger M, Pinard-Bertelletto JP, et al. Heterogeneity in the mutations responsible for X chromosome-linked Kallmann syndrome. *Human molecular genetics* (1993) 2(4):373-7. Epub 1993/04/01. doi: 10.1093/hmg/2.4.373. PubMed PMID: 8504298.

2. Kim HG, Kurth I, Lan F, Meliciani I, Wenzel W, Eom SH, et al. Mutations in CHD7, encoding a chromatin-remodeling protein, cause idiopathic hypogonadotropic hypogonadism and Kallmann syndrome. *American journal of human genetics* (2008) 83(4):511-9. Epub 2008/10/07. doi: 10.1016/j.ajhg.2008.09.005. PubMed PMID: 18834967; PubMed Central PMCID: PMC2561938.

3. Perry JR, Day F, Elks CE, Sulem P, Thompson DJ, Ferreira T, et al. Parent-of-origin-specific allelic associations among 106 genomic loci for age at menarche. *Nature* (2014) 514(7520):92-7. Epub 2014/09/19. doi: 10.1038/nature13545. PubMed PMID: 25231870; PubMed Central PMCID: PMC4185210.

4. Tata B, Huijbregts L, Jacquier S, Csaba Z, Genin E, Meyer V, et al. Haploinsufficiency of Dmxl2, encoding a synaptic protein, causes infertility associated with a loss of GnRH neurons in mouse. *PLoS biology* (2014) 12(9):e1001952. Epub 2014/09/24. doi: 10.1371/journal.pbio.1001952. PubMed PMID: 25248098; PubMed Central PMCID: PMC4172557.

5. Miraoui H, Dwyer AA, Sykiotis GP, Plummer L, Chung W, Feng B, et al. Mutations in FGF17, IL17RD, DUSP6, SPRY4, and FLRT3 are identified in individuals with congenital hypogonadotropic hypogonadism. *American journal of human genetics* (2013) 92(5):725-43. Epub 2013/05/07. doi: 10.1016/j.ajhg.2013.04.008. PubMed PMID: 23643382; PubMed Central PMCID: PMC3644636.

6. Mancini A, Howard SR, Cabrera CP, Barnes MR, David A, Wehkalampi K, et al. EAP1 regulation of GnRH promoter activity is important for human pubertal timing. *Human molecular genetics* (2019) 28(8):1357-68. Epub 2019/01/05. doi: 10.1093/hmg/ddy451. PubMed PMID: 30608578; PubMed Central PMCID: PMC6452208.

7. Corradi A, Croci L, Broccoli V, Zecchini S, Previtali S, Wurst W, et al. Hypogonadotropic hypogonadism and peripheral neuropathy in Ebf2-null mice. *Development* (2003) 130(2):401-10. Epub 2002/12/06. doi: 10.1242/dev.00215. PubMed PMID: 12466206.

8. Kotan LD, Hutchins BI, Ozkan Y, Demirel F, Stoner H, Cheng PJ, et al. Mutations in FEZF1 cause Kallmann syndrome. *American journal of human genetics* (2014) 95(3):326-31. Epub 2014/09/06. doi: 10.1016/j.ajhg.2014.08.006. PubMed PMID: 25192046; PubMed Central PMCID: PMC4157145.

9. Falardeau J, Chung WC, Beenken A, Raivio T, Plummer L, Sidis Y, et al. Decreased FGF8 signaling causes deficiency of gonadotropin-releasing hormone in humans and mice. *The Journal of clinical investigation* (2008) 118(8):2822-31. Epub 2008/07/04. doi: 10.1172/JCI34538. PubMed PMID: 18596921; PubMed Central PMCID: PMC2441855.

10. Dode C, Levilliers J, Dupont JM, De Paepe A, Le Du N, Soussi-Yanicostas N, et al. Loss-of-function mutations in FGFR1 cause autosomal dominant Kallmann syndrome. *Nature genetics* (2003) 33(4):463-5. Epub 2003/03/11. doi: 10.1038/ng1122. PubMed PMID: 12627230.

11. Matthews CH, Borgato S, Beck-Peccoz P, Adams M, Tone Y, Gambino G, et al. Primary amenorrhoea and infertility due to a mutation in the beta-subunit of follicle-stimulating hormone. *Nature genetics* (1993) 5(1):83-6. Epub 1993/09/01. doi: 10.1038/ng0993-83. PubMed PMID: 8220432.

12. Pantel J, Legendre M, Cabrol S, Hilal L, Hajaji Y, Morisset S, et al. Loss of constitutive activity of the growth hormone secretagogue receptor in familial short stature. *The Journal of clinical investigation* (2006) 116(3):760-8. Epub 2006/03/03. doi: 10.1172/JCI25303. PubMed PMID: 16511605; PubMed Central PMCID: PMC1386106.

13. Bouligand J, Ghervan C, Tello JA, Brailly-Tabard S, Salenave S, Chanson P, et al. Isolated familial hypogonadotropic hypogonadism and a GNRH1 mutation. *The New England journal of medicine* (2009) 360(26):2742-8. Epub 2009/06/19. doi: 10.1056/NEJMoa0900136. PubMed PMID: 19535795.

14. de Roux N, Young J, Misrahi M, Genet R, Chanson P, Schaison G, et al. A family with hypogonadotropic hypogonadism and mutations in the gonadotropin-releasing hormone receptor. *The New England journal of medicine* (1997) 337(22):1597-602. Epub 1997/11/27. doi: 10.1056/NEJM199711273372205. PubMed PMID: 9371856.

15. Thomas PQ, Dattani MT, Brickman JM, McNay D, Warne G, Zacharin M, et al. Heterozygous HESX1 mutations associated with isolated congenital pituitary hypoplasia and septo-optic dysplasia. *Human molecular genetics* (2001) 10(1):39-45. Epub 2001/01/04. doi: 10.1093/hmg/10.1.39. PubMed PMID: 11136712.

16. Tornberg J, Sykiotis GP, Keefe K, Plummer L, Hoang X, Hall JE, et al. Heparan sulfate 6-O-sulfotransferase 1, a gene involved in extracellular sugar modifications, is mutated in patients with idiopathic hypogonadotrophic hypogonadism. *Proceedings of the National Academy of Sciences of the United States of America* (2011) 108(28):11524-9. Epub 2011/06/28. doi: 10.1073/pnas.1102284108. PubMed PMID: 21700882; PubMed Central PMCID: PMC3136273.

17. Domene HM, Bengolea SV, Martinez AS, Ropelato MG, Pennisi P, Scaglia P, et al. Deficiency of the circulating insulin-like growth factor system associated with inactivation of the acid-labile subunit gene. *The New England journal of medicine* (2004) 350(6):570-7. Epub 2004/02/06. doi: 10.1056/NEJMoa013100. PubMed PMID: 14762184.

18. Sun Y, Bak B, Schoenmakers N, van Trotsenburg AS, Oostdijk W, Voshol P, et al. Loss-of-function mutations in IGSF1 cause an X-linked syndrome of central hypothyroidism and testicular enlargement. *Nature genetics* (2012) 44(12):1375-81. Epub 2012/11/13. doi: 10.1038/ng.2453. PubMed PMID: 23143598; PubMed Central PMCID: PMC3511587.

19. Howard SR, Guasti L, Ruiz-Babot G, Mancini A, David A, Storr HL, et al. IGSF10 mutations dysregulate gonadotropin-releasing hormone neuronal migration resulting in delayed puberty. *EMBO molecular medicine* (2016) 8(6):626-42. Epub 2016/05/04. doi: 10.15252/emmm.201606250. PubMed PMID: 27137492; PubMed Central PMCID: PMC4888853.

20. Topaloglu AK, Tello JA, Kotan LD, Ozbek MN, Yilmaz MB, Erdogan S, et al. Inactivating KISS1 mutation and hypogonadotropic hypogonadism. *The New England journal of medicine* (2012) 366(7):629-35. Epub 2012/02/18. doi: 10.1056/NEJMoa1111184. PubMed PMID: 22335740.

21. Seminara SB, Messager S, Chatzidaki EE, Thresher RR, Acierno JS, Jr., Shagoury JK, et al. The GPR54 gene as a regulator of puberty. *The New England journal of medicine* (2003) 349(17):1614-27. Epub 2003/10/24. doi: 10.1056/NEJMoa035322. PubMed PMID: 14573733.

22. Mantzoros CS, Flier JS, Rogol AD. A longitudinal assessment of hormonal and physical alterations during normal puberty in boys. V. Rising leptin levels may signal the onset of puberty. *The Journal of clinical endocrinology and metabolism* (1997) 82(4):1066-70. Epub 1997/04/01. doi: 10.1210/jcem.82.4.3878. PubMed PMID: 9100574.

23. Farooqi IS, Wangensteen T, Collins S, Kimber W, Matarese G, Keogh JM, et al. Clinical and molecular genetic spectrum of congenital deficiency of the leptin receptor. *The New England journal of medicine* (2007) 356(3):237-47. Epub 2007/01/19. doi: 10.1056/NEJMoa063988. PubMed PMID: 17229951; PubMed Central PMCID: PMC2670197.

24. Weiss J, Axelrod L, Whitcomb RW, Harris PE, Crowley WF, Jameson JL. Hypogonadism caused by a single amino acid substitution in the beta subunit of luteinizing hormone. *The New England journal of medicine* (1992) 326(3):179-83. Epub 1992/01/16. doi: 10.1056/NEJM199201163260306. PubMed PMID: 1727547.

25. Netchine I, Sobrier ML, Krude H, Schnabel D, Maghnie M, Marcos E, et al. Mutations in LHX3 result in a new syndrome revealed by combined pituitary hormone deficiency. *Nature genetics* (2000) 25(2):182-6. Epub 2000/06/03. doi: 10.1038/76041. PubMed PMID: 10835633.

26. Perry JR, Stolk L, Franceschini N, Lunetta KL, Zhai G, McArdle PF, et al. Meta-analysis of genome-wide association data identifies two loci influencing age at menarche. *Nature genetics* (2009) 41(6):648-50. Epub 2009/05/19. doi: 10.1038/ng.386. PubMed PMID: 19448620; PubMed Central PMCID: PMC2942986.

27. Yeo GS, Farooqi IS, Aminian S, Halsall DJ, Stanhope RG, O'Rahilly S. A frameshift mutation in MC4R associated with dominantly inherited human obesity. *Nature genetics* (1998) 20(2):111-2. Epub 1998/10/15. doi: 10.1038/2404. PubMed PMID: 9771698.

28. Vaisse C, Clement K, Guy-Grand B, Froguel P. A frameshift mutation in human MC4R is associated with a dominant form of obesity. *Nature genetics* (1998) 20(2):113-4. Epub 1998/10/15. doi: 10.1038/2407. PubMed PMID: 9771699.

29. Abreu AP, Dauber A, Macedo DB, Noel SD, Brito VN, Gill JC, et al. Central precocious puberty caused by mutations in the imprinted gene MKRN3. *The New England journal of medicine* (2013) 368(26):2467-75. Epub 2013/06/07. doi: 10.1056/NEJMoa1302160. PubMed PMID: 23738509; PubMed Central PMCID: PMC3808195.

30. Givens ML, Rave-Harel N, Goonewardena VD, Kurotani R, Berdy SE, Swan CH, et al. Developmental regulation of gonadotropin-releasing hormone gene expression by the MSX and DLX homeodomain protein families. *The Journal of biological chemistry* (2005) 280(19):19156-65. Epub 2005/03/04. doi: 10.1074/jbc.M502004200. PubMed PMID: 15743757; PubMed Central PMCID: PMC2932481.

31. Swain A, Zanaria E, Hacker A, Lovell-Badge R, Camerino G. Mouse Dax1 expression is consistent with a role in sex determination as well as in adrenal and hypothalamus function. *Nature genetics* (1996) 12(4):404-9. Epub 1996/04/01. doi: 10.1038/ng0496-404. PubMed PMID: 8630494.

32. Hu SC, Ye J, Fathi AK, Fu X, Huang S, Ning Q, et al. Mutations in NR5A1 and PIN1 associated with idiopathic hypogonadotropic hypogonadism. *Genetics and molecular research : GMR* (2012) 11(4):4575-84. Epub 2012/10/26. doi: 10.4238/2012.October.9.6. PubMed PMID: 23096908.

33. Miura K, Acierno JS, Jr., Seminara SB. Characterization of the human nasal embryonic LHRH factor gene, NELF, and a mutation screening among 65 patients with idiopathic hypogonadotropic hypogonadism (IHH). *Journal of human genetics* (2004) 49(5):265-8. Epub 2004/09/15. doi: 10.1007/s10038-004-0137-4. PubMed PMID: 15362570.

34. Margolin DH, Kousi M, Chan YM, Lim ET, Schmahmann JD, Hadjivassiliou M, et al. Ataxia, dementia, and hypogonadotropism caused by disordered ubiquitination. *The New England journal of medicine* (2013) 368(21):1992-2003. Epub 2013/05/10. doi: 10.1056/NEJMoa1215993. PubMed PMID: 23656588; PubMed Central PMCID: PMC3738065.

35. O'Rahilly S, Gray H, Humphreys PJ, Krook A, Polonsky KS, White A, et al. Brief report: impaired processing of prohormones associated with abnormalities of glucose homeostasis and adrenal function. *The New England journal of medicine* (1995) 333(21):1386-90. Epub 1995/11/23. doi: 10.1056/NEJM199511233332104. PubMed PMID: 7477119.

36. Synofzik M, Gonzalez MA, Lourenco CM, Coutelier M, Haack TB, Rebelo A, et al. PNPLA6 mutations cause Boucher-Neuhauser and Gordon Holmes syndromes as part of a broad neurodegenerative spectrum. *Brain : a journal of neurology* (2014) 137(Pt 1):69-77. Epub 2013/12/21. doi: 10.1093/brain/awt326. PubMed PMID: 24355708; PubMed Central PMCID: PMC3891450.

37. Topaloglu AK, Lomniczi A, Kretzschmar D, Dissen GA, Kotan LD, McArdle CA, et al. Loss-of-function mutations in PNPLA6 encoding neuropathy target esterase underlie pubertal failure and neurological deficits in Gordon Holmes syndrome. *The Journal of clinical endocrinology and metabolism* (2014) 99(10):E2067-75. Epub 2014/07/18. doi: 10.1210/jc.2014-1836. PubMed PMID: 25033069; PubMed Central PMCID: PMC5393493.

38. Saitsu H, Osaka H, Sasaki M, Takanashi J, Hamada K, Yamashita A, et al. Mutations in POLR3A and POLR3B encoding RNA Polymerase III subunits cause an autosomal-recessive hypomyelinating leukoencephalopathy. *American journal of human genetics* (2011) 89(5):644-51. Epub 2011/11/01. doi: 10.1016/j.ajhg.2011.10.003. PubMed PMID: 22036171; PubMed Central PMCID: PMC3213392.

39. Bernard G, Chouery E, Putorti ML, Tetreault M, Takanohashi A, Carosso G, et al. Mutations of POLR3A encoding a catalytic subunit of RNA polymerase Pol III cause a recessive hypomyelinating leukodystrophy. *American journal of human genetics* (2011) 89(3):415-23. Epub 2011/08/23. doi: 10.1016/j.ajhg.2011.07.014. PubMed PMID: 21855841; PubMed Central PMCID: PMC3169829.

40. Tetreault M, Choquet K, Orcesi S, Tonduti D, Balottin U, Teichmann M, et al. Recessive mutations in POLR3B, encoding the second largest subunit of Pol III, cause a rare hypomyelinating leukodystrophy. *American journal of human genetics* (2011) 89(5):652-5. Epub 2011/11/01. doi: 10.1016/j.ajhg.2011.10.006. PubMed PMID: 22036172; PubMed Central PMCID: PMC3213403.

41. Tatsumi K, Miyai K, Notomi T, Kaibe K, Amino N, Mizuno Y, et al. Cretinism with combined hormone deficiency caused by a mutation in the PIT1 gene. *Nature genetics* (1992) 1(1):56-8. Epub 1992/04/01. doi: 10.1038/ng0492-56. PubMed PMID: 1302000.

42. Dode C, Teixeira L, Levilliers J, Fouveaut C, Bouchard P, Kottler ML, et al. Kallmann syndrome: mutations in the genes encoding prokineticin-2 and prokineticin receptor-2. *PLoS genetics* (2006) 2(10):e175. Epub 2006/10/24. doi: 10.1371/journal.pgen.0020175. PubMed PMID: 17054399; PubMed Central PMCID: PMC1617130.

43. Reynaud R, Barlier A, Vallette-Kasic S, Saveanu A, Guillet MP, Simonin G, et al. An uncommon phenotype with familial central hypogonadism caused by a novel PROP1 gene mutant truncated in the transactivation domain. *The Journal of clinical endocrinology and metabolism* (2005) 90(8):4880-7. Epub 2005/06/09. doi: 10.1210/jc.2005-0119. PubMed PMID: 15941866.

44. Young J, Metay C, Bouligand J, Tou B, Francou B, Maione L, et al. SEMA3A deletion in a family with Kallmann syndrome validates the role of semaphorin 3A in human puberty and olfactory system development. *Human reproduction* (2012) 27(5):1460-5. Epub 2012/03/15. doi: 10.1093/humrep/des022. PubMed PMID: 22416012.

45. Cariboni A, Andre V, Chauvet S, Cassatella D, Davidson K, Caramello A, et al. Dysfunctional SEMA3E signaling underlies gonadotropin-releasing hormone neuron deficiency in Kallmann syndrome. *The Journal of clinical investigation* (2015) 125(6):2413-28. Epub 2015/05/20. doi: 10.1172/JCI78448. PubMed PMID: 25985275; PubMed Central PMCID: PMC4497752.

46. Kansakoski J, Fagerholm R, Laitinen EM, Vaaralahti K, Hackman P, Pitteloud N, et al. Mutation screening of SEMA3A and SEMA7A in patients with congenital hypogonadotropic hypogonadism. *Pediatric research* (2014) 75(5):641-4. Epub 2014/02/14. doi: 10.1038/pr.2014.23. PubMed PMID: 24522099.

47. Pingault V, Bodereau V, Baral V, Marcos S, Watanabe Y, Chaoui A, et al. Loss-of-function mutations in SOX10 cause Kallmann syndrome with deafness. *American journal of human genetics* (2013) 92(5):707-24. Epub 2013/05/07. doi: 10.1016/j.ajhg.2013.03.024. PubMed PMID: 23643381; PubMed Central PMCID: PMC3644631.

48. Stark Z, Storen R, Bennetts B, Savarirayan R, Jamieson RV. Isolated hypogonadotropic hypogonadism with SOX2 mutation and anophthalmia/microphthalmia in offspring. *European journal of human genetics : EJHG* (2011) 19(7):753-6. Epub 2011/02/18. doi: 10.1038/ejhg.2011.11. PubMed PMID: 21326281; PubMed Central PMCID: PMC3137491.

49. Solomon NM, Ross SA, Morgan T, Belsky JL, Hol FA, Karnes PS, et al. Array comparative genomic hybridisation analysis of boys with X linked hypopituitarism identifies a 3.9 Mb duplicated critical region at Xq27 containing SOX3. *Journal of medical genetics* (2004) 41(9):669-78. Epub 2004/09/03. doi: 10.1136/jmg.2003.016949. PubMed PMID: 15342697; PubMed Central PMCID: PMC1735898.

50. Kotan LD, Cooper C, Darcan S, Carr IM, Ozen S, Yan Y, et al. Idiopathic Hypogonadotropic Hypogonadism Caused by Inactivating Mutations in SRA1. *Journal of clinical research in pediatric endocrinology* (2016) 8(2):125-34. Epub 2016/04/19. doi: 10.4274/jcrpe.3248. PubMed PMID: 27086651; PubMed Central PMCID: PMC5096466.

51. Jager RJ, Anvret M, Hall K, Scherer G. A human XY female with a frame shift mutation in the candidate testis-determining gene SRY. *Nature* (1990) 348(6300):452-4. Epub 1990/11/29. doi: 10.1038/348452a0. PubMed PMID: 2247151.

52. Shi CH, Schisler JC, Rubel CE, Tan S, Song B, McDonough H, et al. Ataxia and hypogonadism caused by the loss of ubiquitin ligase activity of the U box protein CHIP. *Human molecular genetics* (2014) 23(4):1013-24. Epub 2013/10/12. doi: 10.1093/hmg/ddt497. PubMed PMID: 24113144; PubMed Central PMCID: PMC3900109.

53. Topaloglu AK, Reimann F, Guclu M, Yalin AS, Kotan LD, Porter KM, et al. TAC3 and TACR3 mutations in familial hypogonadotropic hypogonadism reveal a key role for Neurokinin B in the central control of reproduction. *Nature genetics* (2009) 41(3):354-8. Epub 2008/12/17. doi: 10.1038/ng.306. PubMed PMID: 19079066; PubMed Central PMCID: PMC4312696.

54. Satake H, Kawada T. Overview of the primary structure, tissue-distribution, and functions of tachykinins and their receptors. *Current drug targets* (2006) 7(8):963-74. Epub 2006/08/22. doi: 10.2174/138945006778019273. PubMed PMID: 16918325.

55. Kim HG, Ahn JW, Kurth I, Ullmann R, Kim HT, Kulharya A, et al. WDR11, a WD protein that interacts with transcription factor EMX1, is mutated in idiopathic hypogonadotropic hypogonadism and Kallmann syndrome. *American journal of human genetics* (2010) 87(4):465-79. Epub 2010/10/05. doi: 10.1016/j.ajhg.2010.08.018. PubMed PMID: 20887964; PubMed Central PMCID: PMC2948809.
